# *GhTIE1* Regulates Branching Through Modulating the Transcriptional Activity of TCPs in Cotton and *Arabidopsis*

**DOI:** 10.3389/fpls.2019.01348

**Published:** 2019-10-28

**Authors:** Yangyang Diao, Jingjing Zhan, Yanyan Zhao, Lisen Liu, Peipei Liu, Xi Wei, Yanpeng Ding, Muhammad Sajjad, Wei Hu, Peng Wang, Xiaoyang Ge

**Affiliations:** ^1^State Key Laboratory of Cotton Biology, Institute of Cotton Research of Chinese Academy of Agricultural Sciences, Anyang, China; ^2^State Key Laboratory of Cotton Biology (Hebei Base), College of Agronomy, Hebei Agricultural University, Baoding, China

**Keywords:** *TIE1*, shoot branching, *TCP*, plant type, cotton, *Arabidopsis*

## Abstract

Transcription factors (TFs) and transcriptional regulators are important switches in transcriptional networks. In recent years, the transcriptional regulator TIE1 (TCP interactor containing EAR motif protein 1) was identified as a nuclear transcriptional repressor which regulates leaf development and controls branch development. However, the function and regulatory network of *GhTIE1* has not been studied in cotton. Here, we demonstrated that *GhTIE1* is functionally conserved in controlling shoot branching in cotton and *Arabidopsis*. Overexpression of *GhTIE1* in *Arabidopsis* leads to higher bud vigor and more branches, while silencing *GhTIE1* in cotton reduced bud activity and increased branching inhibition. Yeast two-hybrid (Y2H) and bimolecular fluorescence complementation (BiFC) assays showed that GhTIE1 directly interacted with subclass II TCPs (GhBRC1, GhBRC2, and GhTCP13) *in vivo* and *in vitro*. Overexpression of *GhBRC1*, *GhBRC2*, and *GhTCP13* in mutant *brc1*-2 partially rescued the mutant phenotype and decreased the number of branches, showing that these TCPs are functionally redundant in controlling branching. A transient dual-luciferase reporter assay indicated that GhTIE1 repressed the protein activity of GhBRC1 and GhTCP13, and thereby decreased the expression of their target gene *GhHB21*. Gene expression level analysis in *GhTIE1*-overexpressed and silenced plants also proved that *GhTIE1* regulated shoot branching *via* repressing the activity of *BRC1*, *HB21*, *HB40*, and *HB53*. Our data reveals that shoot branching can be controlled *via* modulation of the activity of the *TIE1* and *TCP* proteins and provides a theoretical basis for cultivating cotton varieties with ideal plant types.

## Introduction

Branch development plays a decisive role in controlling aboveground plants, and the aboveground parts of crops are closely related to growth and yield (Kebrom et al., 2012). The development of shoots begins with axillary meristem and then develops into small shoots (McSteen and Leyser, 2005; Pasternak et al., 2019; Wang et al., 2019). The regulatory network consists of plant hormones and transcription factors (TFs) that can break the dormancy of small shoots and eventually develop into lateral branches (Rameau et al., 2015; Wang and Jiao, 2018). Cotton is an infinitely growing crop with a complex branching pattern consisting of fruit branches, vegetative shoots, and axillary buds. Developing and cultivating the ideal plant type not only affects cotton production but also saves a lot of labor. Therefore, the study of cotton branching control provides an important theoretical basis for improvement of cotton plants.

At present, with increased research on plant branch development, *TCP* (*TEOSINTE BRANCHED1* (*TB1*) from maize, *BRC1* from *Arabidopsis*, and *PROLIFERATING CELL FACTOR* from rice) family TFs regulating branch development have been successively discovered. *TB1* was first found to be a key gene controlling maize branching (Dong et al., 2017); subsequently, the homologous genes of *TB1* in other species, such as rice *FINE CULM* 1/*OsTB1* (Takeda et al., 2010), *Arabidopsis* *BRANCHED1* (*BRC1*) (Aguilar-Martínez et al., 2007), pea *PsBRC1* (Nils et al., 2012), potato *BRANCHED1* (Nicolas et al., 2015), and tomato *BRC1*-like genes (Martı´n-Trillo et al., 2011) were identified. Similar to corn *TB1*, overexpression of these genes inhibits branching, while loss-of-function mutations lead to increased branching (Gaudin et al., 2014; Dong et al., 2017). Transgenic rice plants overexpressing *OsTB1* showed significantly reduced branching (Takeda et al., 2010). The *Arabidopsis thaliana* *BRC1* gene was mainly expressed in axillary buds (axillary meristem, bud primordium, and nourishing vascular tissue), and the expression level decreased with the growth of buds (Finlayson, 2007). The loss-of-function *BRC1* mutant *brc1*-2 is characterized by an accelerated onset of axillary meristems with faster shoot development and more branches. Related studies have also demonstrated that *BRC1* is an integrator that controls the internal and external factors of bud activity (Aguilar-Martínez et al., 2007). Hormone strigolactone positively regulates *BRC1* at the transcriptional level and inhibits plant branching (Chevalier et al., 2014; Minakuchi et al., 2010). Cytokinin negatively regulates *BRC1* expression, thereby promoting branch growth in rice and peas (Arite et al., 2010; Takeda et al., 2010; Dun et al., 2012; Nils et al., 2012).

In recent years, the network consisting of upstream genes regulating *BRC1* and downstream targets of *BRC1* has been revealed. Rice TF *IPA1* acts as an upstream gene, which promotes the expression of *OsTB1* by directly binding to the promoter region (Lu et al., 2013). *BRC1* downstream target genes were initially discovered, including three HD-ZIP TFs—*HOMEOBOX* protein (*HB*) 21, *HB40*, and *HB53*—which are directly regulated by *BRC1* in *Arabidopsis* (González-Grandío et al., 2017). Recently, a new transcriptional regulator, TIE1 (TCP interactor containing EAR motif protein 1), was identified to control *Arabidopsis* branching by controlling the activity of *BRC1* protein and then repressing the expression of *BRC1* target genes *HB21*, *HB40*, and *HB53*. In the development of branching, overexpression of *TIE1* results in higher shoot vigor and more branches, while disruption of *TIE1* reduces shoot activity and increases branch inhibition (Tao et al., 2013; Yang et al., 2018).

Studies have shown that the developmental control of branches in different species is both conserved and specific (Wang et al., 2018). There are, however, no studies on the function of *GhTIE1* in regulating cotton branching. Our research indicates that *GhTIE1* is a transcriptional repressor that is expressed in both the nucleus and cytoplasm. *Arabidopsis* overexpressing *GhTIE1* produces more branches, and gene silencing of *GhTIE1* leads to decreased branching in cotton. *GhBRC1*, *GhBRC2*, and *GhTCP13* interact with *GhTIE1* *in vitro* and *in vivo*. Overexpression of *GhBRC1*, *GhBRC2*, and *GhTCP13* in the mutant *brc1*-2 partially rescued the branching phenotype, suggesting their redundant function in controlling branching. Expression level analysis supported that *GhTIE1* indirectly inhibits the expression of *BRC1* target genes *HB21*, *HB40*, and *HB53* by inhibiting the activity of *BRC1*. The results of our study show that *GhTIE1* is functionally conserved in controlling branching in cotton and *Arabidopsis*, and shoot branching was controlled by modulating the activity of the *TIE1* and *TCP* proteins.

## Materials and Methods

### Plant Materials and Growth Conditions

The cotton materials used in this study were *Gossypium hirsutum* cultivar ZM24. Cotton seeds were immersed in sterile H_2_O for 24 h, then planted in mixed soils including trophic soils and vermiculite (1:1, *v*/*v*), and grown with regular watering under adjusted conditions of 27/20°C, 14/10 h, and humidity of 75%.

*Arabidopsis* *thaliana* ecotype Columbia-0 (Col-0) was used in this study. The mutant *brc1*-2 was obtained from the laboratory of Dr Genji Qin. The *Arabidopsis* seeds were sterilized by commercially diluted (1:1, *v*/*v*) NaOCl, followed by several rinses with sterile water. Germination was carried out on sterile Murashige and Skoog (MS) medium. After 7 days, seedlings were transferred to soil and grown at 22°C under long-day conditions (16-h light and 8-h dark, 70% relative humidity).

### Gene Expression Assays

We obtained the apical new tissue of cotton, including stem, leaves, and axillary buds. For *A. thaliana*, we used the rosette disc with a bit of the stem and then extracted RNA using the RNAprep Pure Plant Kit (Tiangen). We then used the PrimeScript RT reagent Kit with gDNA Eraser (Takara) to produce cDNAs.

For RT-PCR, the following parameters were used: 94°C for 5 min, 27 cycles at 98°C for 15 s, 60°C for 15 s, and 68°C for 30 s. Diluted cDNA was used for quantitative RT-PCR (qRT-PCR) with SYBR Premix Ex Taq (Tli RNaseH Plus, TAKARA) on an ABI 7900 qRT-PCR System (Applied Biosystems). A three-step method was used with the following PCR conditions: 40 cycles at 95°C for 30 s, 95°C for 5 s, and 60°C for 30 s. We checked the dissociation curves of each reaction and used the cycle threshold (CT) 2^–∆∆Ct^ method to calculate the expression level of each target gene (Livak and Schmittqen, 2001). Each reaction was performed with at least three biological replicates. All the primers used in this study are listed in **Supplementary Table 1**. All the qPCR data are showed in **Supplementary Table 2**.

### Construction of Overexpression and Virus-Induced Gene Silencing Vector

In our study, we constructed all vectors using a one-step cloning method. This method uses a primer with a homologous arm and a double restriction site to amplify the gene fragment, which is then ligated to the vector with the same homology arm and restriction site. All vectors were converted to *Agrobacterium tumefaciens* strain GV3101 using the freeze–thaw method.

To construct the overexpression vector, the primers 35S-*TIE1*-F/R and 35S-*BRC1*-F/R (**Supplementary Table 1**) were used to ligate the amplified gene to a pCAMBIA2300 vector. *A. thaliana* inflorescences were immersed in a suspension of *Agrobacterium* to obtain transgenic *A. thaliana* (Clough and Bent, 1998).

For the VIGS experiments, the primers V*TIE1*-F/R and V*BRC1*-F/R (**Supplementary Table 1**) were used. A conserved 251-bp fragment of *GhTIE1a and GhTIE1d* was cloned into a pCLCrVA vector to generate pCLCrV::*GhTIE1*, and pCLCrV-CHL1 was used as a positive control. The constructs were then transformed to GV3101. The cotyledons of 7-day-old ZM24 cotton seedlings were injected with equal amounts of *Agrobacterium* expressing the CLCrV vectors. Two-week-old cotton leaves were sampled for real-time PCR to check the interference efficiency.

### Subcellular Localization

Analysis of the amino acid sequence of *GhTIE1* identified the nuclear signal peptide. In order to verify whether it is expressed in the nucleus, we first cloned the gene with the primer QBV3-*GhTIE1*-F/R, then ligated it to the vector QBV3, and finally constructed the vector into the YFP fluorescent tag-containing vector pEG101 by an LR reaction to obtain the fused expression vector *GhTIE1*-YFP (Qian et al., 2013).

To confirm the cellular localization of *GhTIE1*, we injected tobacco leaves with *Agrobacterium* containing the *GhTIE1*-YFP vector to detect transient expression. After 48 h of inoculation, the infiltrated *Nicotiana benthamiana* leaves were observed by a laser scanning confocal microscope (OLYMPUS FV1200 confocal microscope). Localization of *GhBRC1*, *GhBRC2*, and *GhTCP13* was obtained using the same method. Sequences of the genes in this study were retrieved from the CottonGen database (https://www.cottongen.org/) (Zhang et al., 2015).

### Yeast Two-Hybrid Assays

To examine the interaction of *TIE1* with *BRC1*, *BRC2*, and *GhTCP13*, we amplified the coding sequences for *TIE1* and *BRC1* and for *BRC2* and *GhTCP13* using BD*TIE1* F/R, AD*BRC1*-F/R, AD*BRC2*-F/R, and AD*TCP13*-F/R, respectively (**Supplementary Table 1**). The bait structure BD-*TIE1* was cloned into the BD-pGBKT7 vector, and the prey structures AD-*BRC1*, AD-*BRC2*, and AD-*GhTCP13* were ligated to the AD-pGADT7 vector. The bait structure and each prey were co-transformed into yeast strain AH109. Glucose-free medium (SD/-2) without Leu and Trp and glucose-free medium (SD/-4) without Leu, Trp, His, and Ade were used for culturing all transformed yeast cells.

### BiFC Assays and Luc Assay

The vectors pXY104 and pXY106 were used to produce constructs for the bimolecular fluorescence complementation (BiFC) assays, and they carried fragments encoding the C- and N-terminal halves of YFP (cYFP and nYFP), respectively (Yu et al., 2008). The cDNA fragments encoding *GhBRC1* were fused to the fragment encoding the C-terminus of YFP, and the fragments encoding *GhTIE1* were fused to the fragment encoding the N-terminus of YFP, then transformed into *Agrobacterium* strain GV3101. GV3101 cultures harboring constructs expressing nYFP fusion proteins and cYFP fusion proteins were mixed at a ratio of 1:1 and introduced into *N. benthamiana* leaves through infiltration. YFP signals were detected by confocal microscopy as described above. For the luciferase (LUC) assay, a 1,500-bp promoter region of *GhHB21* was constructed into a vector pGWB435 containing a Luc reporter gene to construct the reporter *GhHB21*pro-LUC. *GhHB21*pro-LUC and 35S-pCAMBIA2300-GFP were co-infiltrated into *N. benthamiana* leaves as a positive control; *GhHB21*pro-LUC, 35S-pCAMBIA2300-*GhBRC1*, and 35S-pCAMBIA2300-*GhTIE1* were co-infiltrated into *N. benthamiana* leaves to confirm whether *GhTIE1* represses the *GhBRC1* activity; and 35S-pCAMBIA2300-Gh*TCP*13, *GhHB21*pro-LUC, and 35S-pCAMBIA2300-*GhTIE1* were co-infiltrated into *N. benthamiana* leaves to detect the interaction between *TIE1* and *BRC1* (Chen et al., 2008). The plants were placed in the dark for 12 h, followed by 48 h in a growth chamber under normal conditions. The infiltrated *N. benthamiana* leaves were sprayed with luciferin (100 mM) and kept in the dark for 10 min. The leaves were observed under a low-light cooled charge-coupled device (CCD) imaging apparatus Lumazone_1300B (Roper Bioscience) and then photos were taken. Quantitative analysis was done using a Tanon 5200 Multi chemiluminescent imaging system.

## Results

### Sequence Analysis and Subcellular Localization of *GhTIE1a* and *GhTIE1d*

**Figure 1:**
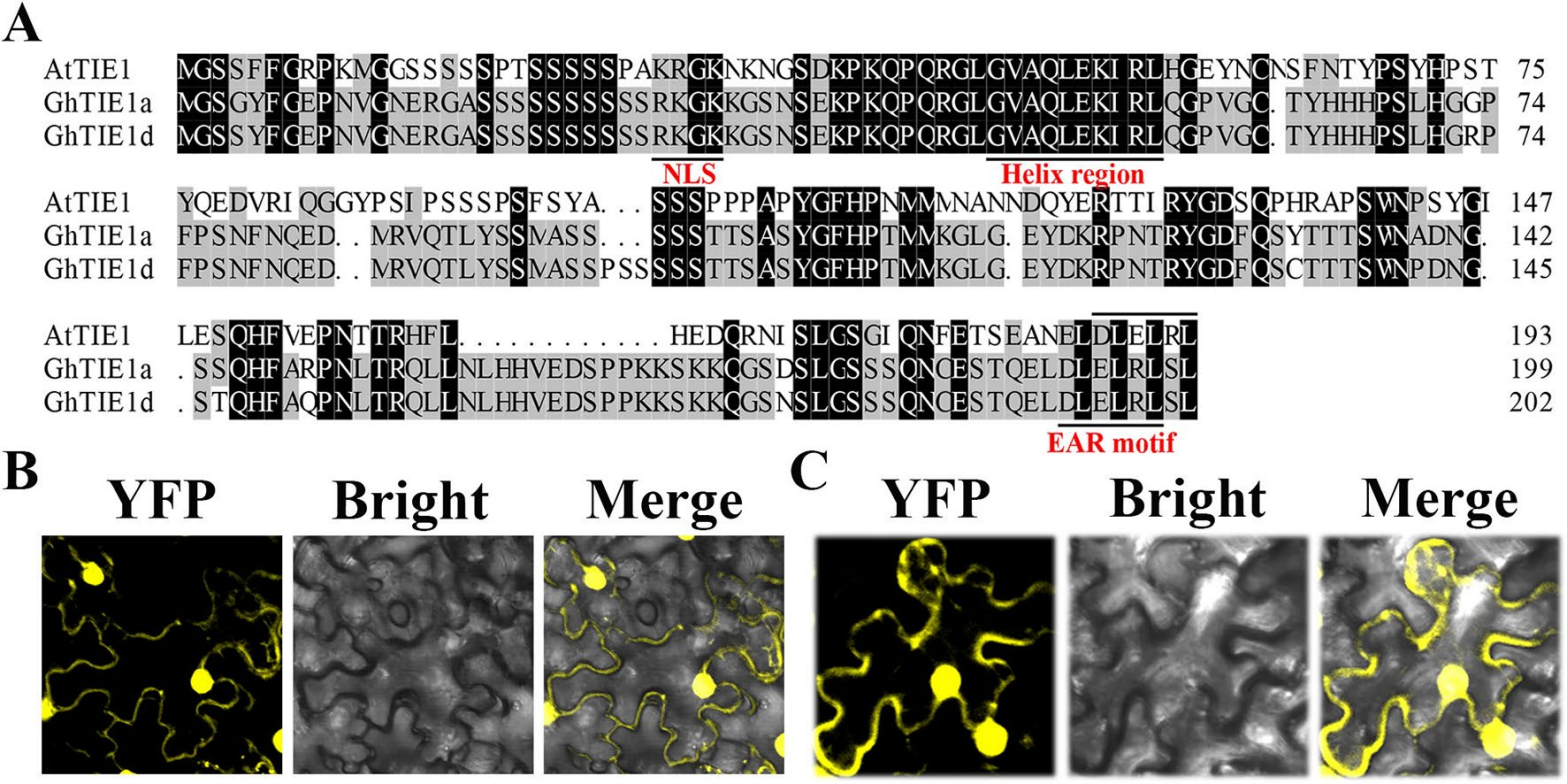
Sequence analysis and subcellular localization of *GhTIE1*. **(A)** Sequence alignment and domain analysis of *TIE1* in *Arabidopsis* and upland cotton. Amino acid sequence alignment of *AtTIE1* (At4g28840), *GhTIE1d* (GH_D05G0259), and *GhTIE1a* (GH_A05G0254). They all contain a nuclear localization signal, a helix region, and an EAR motif. **(B)** *GhTIE1d* and **(C)** *GhTIE1a* are localized in the nucleus and the cytoplasm, mostly in the nucleus.

Using the *AtTIE1* amino acid sequence as a query sequence, we searched the *G*. *hirsutum* protein database using the blastp program (https://www.cottongen.org/) to obtain two homologous genes located in At (GH_A05G0254) and Dt (GH_D05G0259) subgenomes, respectively, named *GhTIE1a* and *GhTIE1d* (**Figure 1A**). We chose GhTIE1a, GhTIE1d, AtTIE1(At4G28840.1), AtTIE2(At2G20080), AtTIE3(At1G29010), and AtTIE4(At2G34010) to make a phylogenetic tree. It showed that GhTIE1 are closer to AtTIE1 than the other AtTIE genes (**Supplementary Figure 1**). We cloned *GhTIE1a* and *GhTIE1d* from upland cotton; *GhTIE1a* and *GhTIE1d* encode proteins containing 199 and 202 amino acids, respectively. The sequence alignment showed that the similarities of *GhTIE1a* and *GhTIE1d* protein sequences to *AtTIE1* were 45.95% and 45.74%, respectively. The similarity between *GhTIE1a* and *GhTIE1d* protein sequences reached 96.48%. Despite the low similarity of TIE1 between *Arabidopsis* and cotton, some of the conserved domains of the two *GhTIE1*s are highly similar to *AtTIE1*: the N-terminal region contains a helical region (between residues 46 and 55), and the C-terminal region contains a typical EAR motif sequence (DLELRL), showing that *GhTIE1a* and *GhTIE1d* may possess a similar function to *AtTIE1* as a transcriptional repressor (**Figure 1A**) (Yang et al., 2018). The putative nuclear localization signal (KRGK) was located at the N-terminus of the *GhTIE1a* and *GhTIE1d* proteins, suggesting that *GhTIE1a* and *GhTIE1d* might be localized in the nucleus (Dingwall et al., 1988; Ohta et al., 2001). *GhTIE1*-YFP was infiltrated into tobacco leaves to detect transient expression, and the results showed that *GhTIE1a* and *GhTIE1d* proteins were localized in the nucleus and the cytoplasm, but this was more pronounced in the nucleus (**Figures 1B, C**).

### Overexpression of *GhTIE1a* and *GhTIE1d* in *Arabidopsis* Promotes Shoot Branching

**Figure 2:**
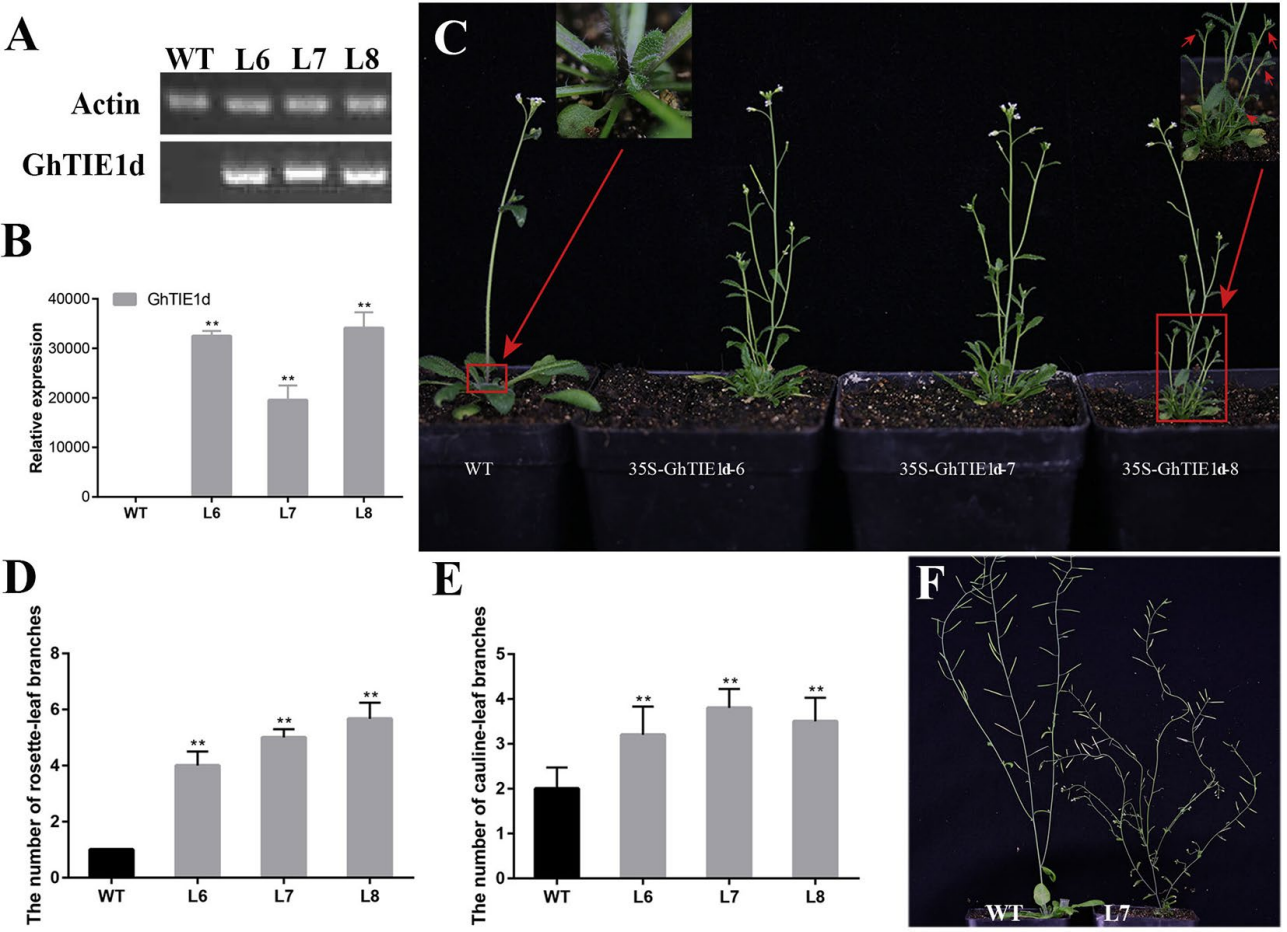
Overexpression of *GhTIE1d* causes excessive branching. **(A)** *GhTIE1d* was not expressed in WT, but was highly expressed in three overexpressed lines based on semi-quantitative PCR and **(B)** qRT-PCR. **(C)** Branching phenotypes of 35-day-old WT plants and three overexpressed 35S-*GhTIE1d* lines. Overexpressing *GhTIE1d* in *Arabidopsis* produced more branches than in wild-type plants. **(D)** Quantitative analysis of rosette-leaf branches in 35-day-old wild-type and three overexpressed lines. **(E)** Quantitative analysis of cauline-leaf branches in 80-day-old wild-type and three overexpressed lines. **(F)** Cauline-leaf branching phenotypes of 80-day-old WT plants and overexpressed 35S-*GhTIE1d*-7 lines. Overexpressing *GhTIE1d* in *Arabidopsis* produced more cauline-leaf branches than in wild-type plants. The rosette-leaf branches have been removed for better visualization of the main stem. The rosette-leaf branches and cauline-leaf branches of the overexpressed lines are significantly increased. L6, L7, and L8 are the three different overexpressed lines, 35S-*GhTIE1d*-6, 35S-*GhTIE1d*-7, and 35S-*GhTIE1d*-8, respectively. *WT* wild type (**p < 0.01). Data represent the mean ± SD from three biological replicates.

*GhTIE1* and *AtTIE1* share conserved domains including an EAR motif and a helix region and are both mainly located in the nucleus, indicating that *GhTIE1* may have a similar function as *AtTIE1* in controlling shoot branching. To confirm the function of *GhTIE1*, overexpressed vectors 35S-*GhTIE1a* and 35S-*GhTIE1d* were constructed to transform *Arabidopsis*. These overexpression lines with 35S-*GhTIE1a* and 35S-*GhTIE1d* both showed increased rosette-leaf branching and cauline-leaf branching (**Figure 2** and **Supplementary Figure 2**), suggesting that *GhTIE1a* and *GhTIE1d* are functionally redundant in regulating shoot branching.

Considering the functional redundancy of *GhTIE1a* and *GhTIE1d*, only *GhTIE1d*-overexpressed *Arabidopsis* plants were further analyzed. Three homozygous lines—35S-*GhTIE1d*-6, 35S-*GhTIE1d*-7, and 35S-*GhTIE1d*-8, were selected to analyze the expression level and shoot branch phenotype. *GhTIE1d* was highly expressed, as confirmed by semi-quantitative PCR and qRT-PCR in these homozygous lines (**Figures 2A, B**). The shoot branch including a cauline-leaf branch and a rosette-leaf branch was obviously increased in Gh*TIE1d*-overexpressed *Arabidopsis* compared to that of wild type (WT) in 35-day-old seedlings, where there was no observed rosette-leaf branch in WT. Most rosette-leaf buds remained small or dormant. Almost all the rosette-leaf buds in overexpressed (OE) lines grew out to form rosette-leaf branches, and more than four rosette-leaf branches were observed in OE lines (**Figures 2C, D**). Similarly, the cauline-leaf branch was obviously increased in OE lines compared to WT, where only one cauline-leaf branch was initially grown in WT but more than two cauline-leaf branches were observed in OE lines in 35-day-old seedlings (**Figure 2C**), suggesting that overexpression of *GhTIE1d* promoted axillary bud activity and the initiation and growth of cauline-leaf branch. In later reproductive stages, the cauline-leaf branch number was also increased in OE lines compared to WT in 80-day-old seedlings, where there were only about two cauline-leaf branches in WT but more than three cauline-leaf branches in OE lines (**Figures 2E, F**). These results showed that *GhTIE1* positively regulates shoot branching growth vigor and shoot branch number.

### Silencing *GhTIE1* in Cotton Seriously Decreases Shoot Branching Number

**Figure 3:**
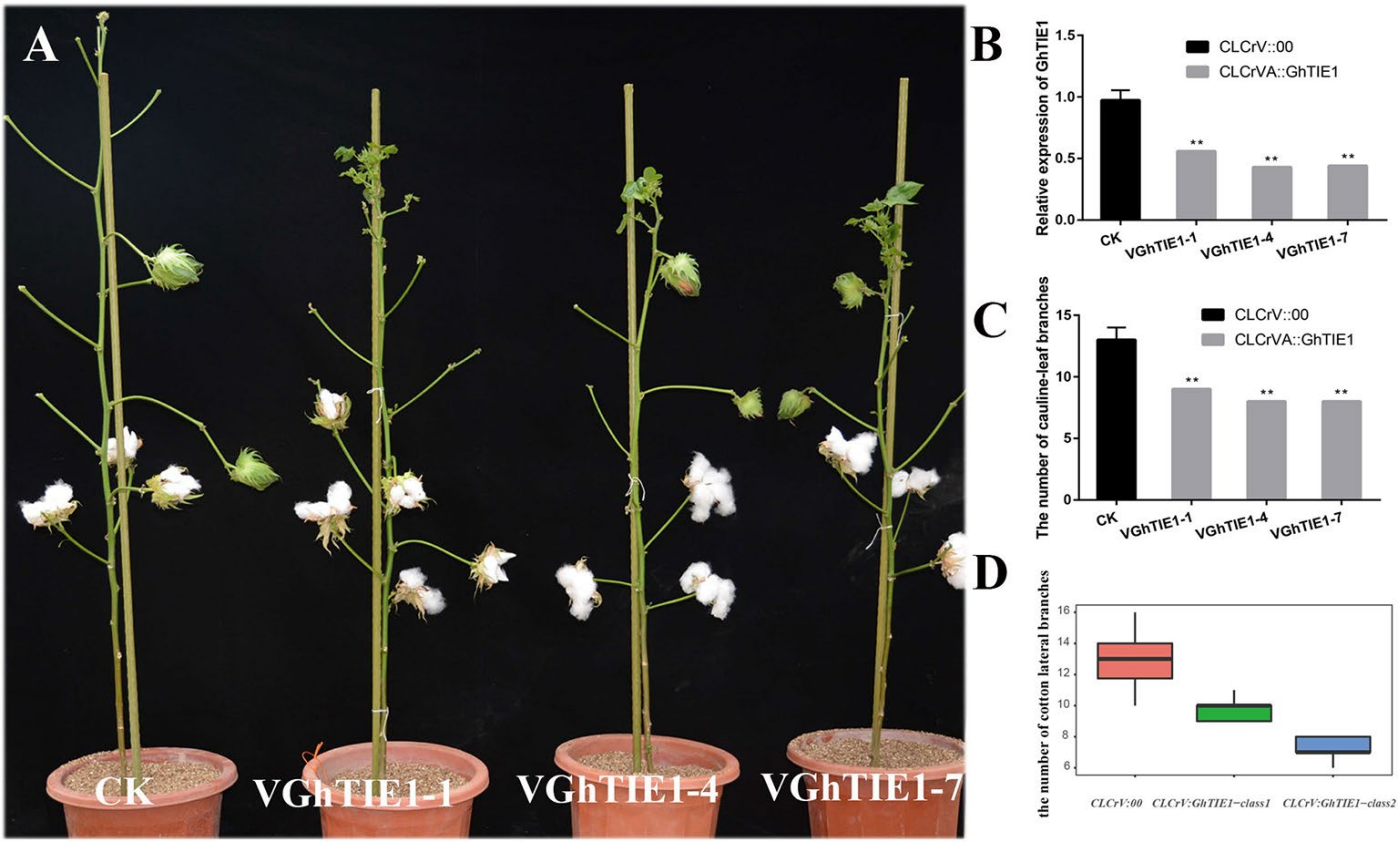
Silencing of *GhTIE1* in cotton seriously decreases shoot branching. **(A)** Branching phenotypes of cotton grown for 4 months of control (CLCrV:00) and Gh*TIE1*-silenced (V*GhTIE1*-1, V*GhTIE1*-4, and V*GhTIE1*-7) plants. **(B)** Analysis of the *GhTIE1* gene expression in CK and VIGS plants by qRT-PCR in three different lines: V*GhTIE1*-1, V*GhTIE1*-4, and V*GhTIE1*-7. **(C)** Quantitative analysis of branches of CLCrV:00 and *GhTIE1*-silenced plants (**p < 0.01). V*GhTIE1*-1, V*GhTIE1*-4, and V*GhTIE1*-7 are the three different silenced cotton plants. CK, the empty vector CLCrV:00. **(D)** Analysis of the number of branches in 20 different CLCrV:00 and Gh*TIE1*-silenced cotton plants. CLCrV : GhTIE1-class1, the cotton plants with a silencing efficiency less than 50%; CLCrV : GhTIE1-class2, the cotton plants with a silencing efficiency more than 50%. Data represent the mean ± SD from three biological replicates.

*GhTIE1a* and *GhTIE1d* are highly functionally redundant in regulating *Arabidopsis* shoot branching. Considering the functional redundancy caused by two copies of *GhTIE1*, the highly conserved CDS fragment between *GhTIE1a* and *GhTIE1d* was cloned into a CLCrV vector to simultaneously silence these two copies. We generated a CLCrV : *GhTIE1* construct to transform the cotyledon of cotton. Under the constant conditions of a 16-h light/8-h dark cycle at 25°C, most of the control plants produced about 13 branches, while almost all cotton plants with the *GhTIE1* silenced showed approximately nine branches (**Supplementary Table 3**). Three *GhTIE1*-silenced plants with a relatively higher gene silencing efficiency, where the *GhTIE1* expression level was less than 50%, exhibited less than nine shoot branches (**Figures 3A–C**). Based on the *GhTIE1* inferring efficiency, the *GhTIE1*-silenced plants were grouped into two classes, where the shoot branch number was less than 9 when the silencing efficiency was more than 50%. In contrast, the shoot branch number was between 10 and 11 when the silencing efficiency was less than 50% (**Figure 3D**). These results indicate that, like *AtTIE1*, *GhTIE1* positively regulates shoot branching number in cotton.

### *GhTIE1* Interacts With *GhBRC1*, *GhBRC2*, and *GhTCP13* and Represses Their Activity

**Figure 4:**
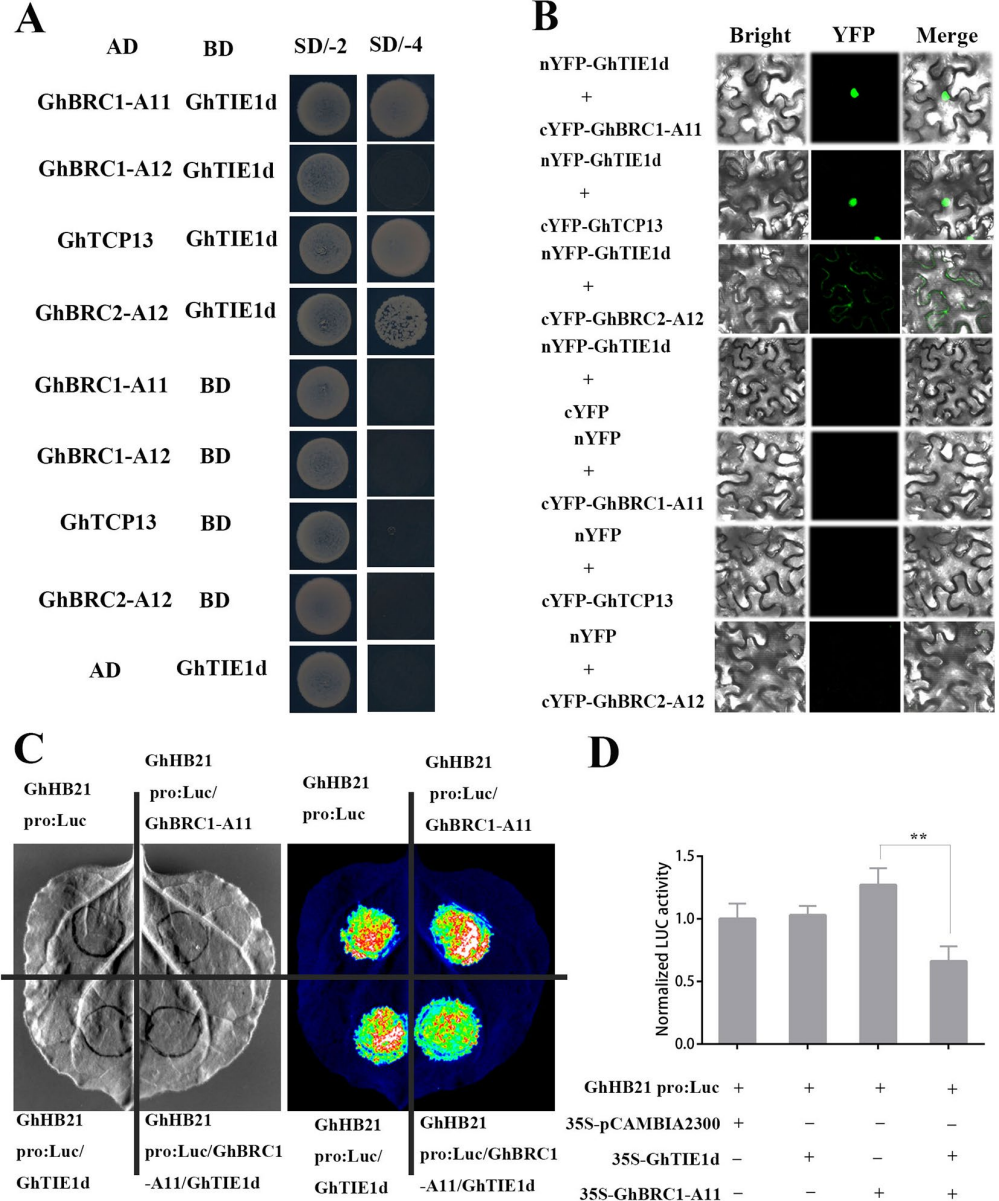
*GhTIE1* interacts with *GhBRC1*, *GhBRC2*, and *GhTCP13* and represses their activity. **(A)** Yeast two-hybrid assays of *GhTIE1d* (GH_D05G0259) with *GhBRC1-A11* (GH_A11G0062), *GhBRC1-A12* (GH_A12G2854), *GhBRC2-A12* (GH_A12G1898), and *GhTCP13* (GH_A05G3929). **(B)** BiFC assay proved the interaction of *GhTIE1* with *GhBRC1-A11*, *GhBRC2-A12*, and *GhTCP13*. **(C)** The promoter of the *GhHB21* gene fused to LUC was used as a reporter for the transactivation assay. *GhTIE1* inhibited *GhHB21* expression *via* interaction with *GhBRC1* (GH_A11G0062). **(D)** LUC activity was decreased when *GhTIE1d*, *GhBRC1-A11*, and *GhHB21* pro:Luc were co-infiltrated into *Nicotiana benthamiana* leaves (**p < 0.01). SD/-2 means SD-Leu-Trp; SD/-4 means SD-Leu-Trp-His-Ade. Data represent the mean ± SD from three biological replicates.

In *Arabidopsis*, *AtTIE1* interacted with *AtBRC1* and inhibited *AtBRC1* activity to activate bud activity (Yang et al., 2018). To reveal the molecular mechanism of *GhTIE1* regulating shoot branching in cotton, cotton axillary buds, shoot branches, and leaves were collected to construct a library. The *GhTIE1* interacting proteins were screened *via* the yeast two-hybrid system. Several class II TCP genes, including *GhBRC1* (*TCP*18), *GhTCP13*, and *GhBRC2* (*TCP*12), potentially interacted with *GhTIE1*. The protein blast results and phylogenetic analysis showed that four *GhBRC1* proteins were homologous to *AtBRC1* and two *GhBRC2* proteins were homologous to *AtBRC2* (**Supplementary Figures 3** and **4**). To further verify the interaction between GhTIE1d and GhTCPs, the full-length CDS sequence of candidate *GhTCP*s were cloned into AD vectors to perform yeast two-hybrid assays. The results confirmed that two copies of *GhBRC1* (GH_D11G0067, GH_A11G0062, GH_D12G2879, and GH_A12G2854) and *GhBRC2* (GH_A12G1898 and GH_D12G1898) and one copy of *GhTCP13* (GH_A05G3929) interacted with GhTIE1d (**Figure 4A** and **Supplementary Figure 5A**). To study the interaction of the above proteins *in vivo*, BiFC assays were performed. Two copies of *GhBRC1*, two copies of *GhBRC2*, and one copy of *GhTCP13* were interacted with *GhTIE1* in the nucleus, but GhBRC2 was shown to interact with GhTIE1d in the cell cytoplasm (**Figure 4B** and **Supplementary Figure 5B**). Consistent with the BiFC results, *GhBRC1*, *GhBRC2*, and *GhTCP13* were all localized in the nucleus and cytoplasm (**Supplementary Figure 6**).

*AtHB21*, *AtHB40*, and *AtHB53* were the direct targets of AtBRC1, and AtTIE1 interacted with AtBRC1 to control the shoot branching *via* repressing *AtHB21*, *AtHB40*, and *AtHB53* expression (Yang et al., 2018). To confirm whether GhTIE1 represses GhBRC1 transcriptional activity, the *AtHB21* homologous gene *GhHB21* was selected, and the 1,500-bp promoter region upstream of *GhHB21* was fused to LUCIFERASE (LUC) to construct the reporter construct *GhHB21*pro-LUC. Then, 35S-GFP as control, 35S-*GhBRC1*, and 35S-*GhTIE1* were co-infiltrated into *N. benthamiana* leaves with *GhHB21*pro-LUC. LUC activity analysis indicated that *GhBRC1* directly activated the *GhHB21*pro-LUC. In contrast, when *GhHB21*pro-LUC and 35S-*GhBRC1* were co-infiltrated with 35S-*GhTIE1*, the activity of *GhHB21*pro-LUC was inhibited, showing that GhTIE1 represses GhBRC1 transcriptional activity (**Figures 4C, D**). Similarly, in transient expression of tobacco leaves, GhTIE1 also inhibited GhTCP13 transcriptional activity, thereby repressing *GhHB21* expression (**Supplementary Figure 7**). These results showed that *GhTIE1* controlled shoot branching *via* repressing *GhBRC1*, *GhBRC2*, and *GhTCP13* activity, and the *GhTIE1* and *GhBRC1* interaction model was conserved as described in *Arabidopsis*, where *AtTIE1* interacted with *AtBRC1* and repressed *BRC1* protein activity. Moreover, the interactions of *GhTIE1* and *GhBRC2* and of *GhTIE1* and *GhTCP13* were specific in cotton and not reported in *Arabidopsis*, suggesting that the *GhTIE1* regulatory network was more complex than *AtTIE1* in regulating branching.

### *GhBRC1* and *GhBRC2* are Functionally Conserved in Regulating Shoot Branching

**Figure 5:**
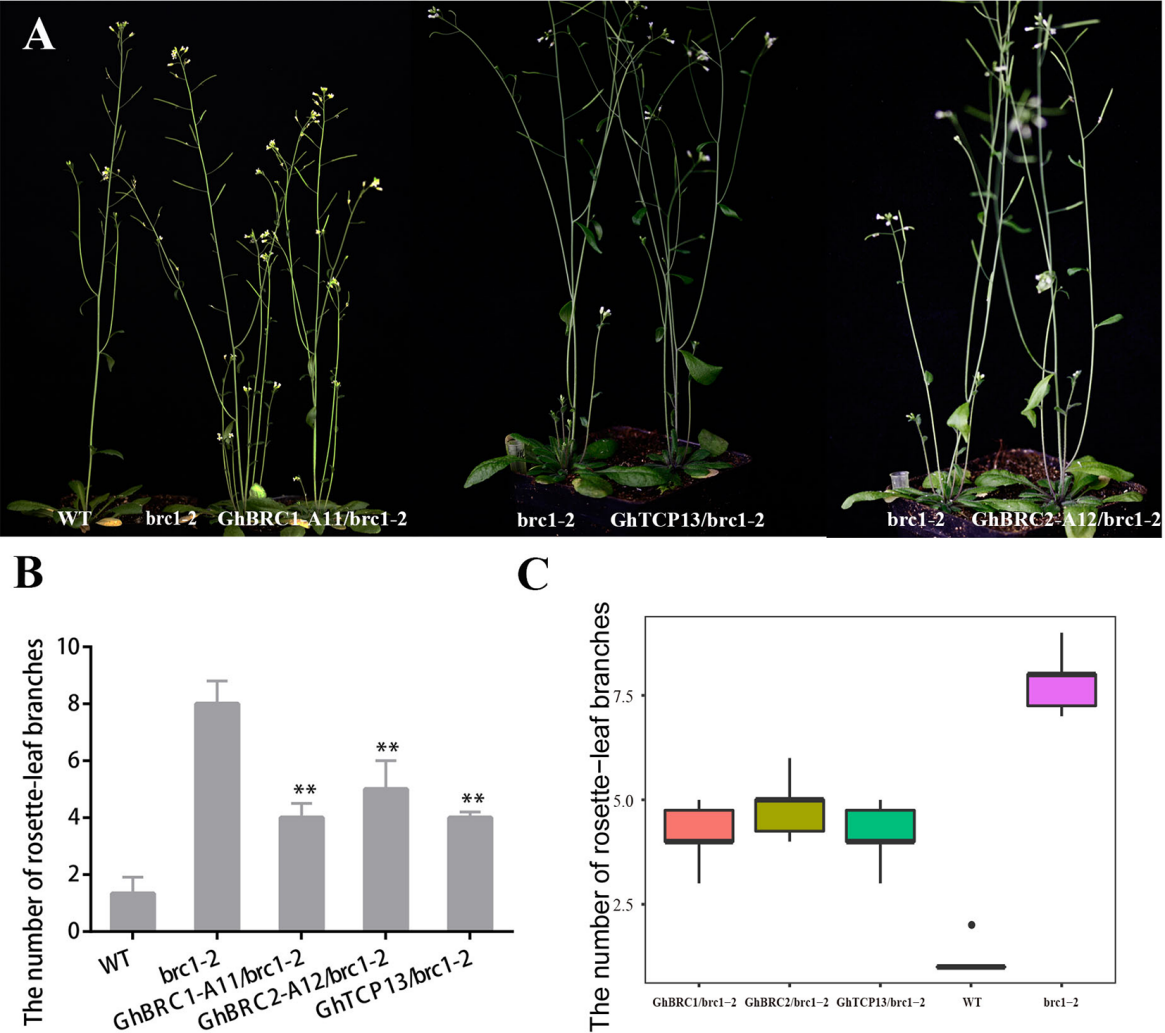
Overexpression of *GhBRC1*, *GhBRC2*, or *GhTCP13* partially restores the phenotype of branching in *Arabidopsis* mutant *brc1*-2. **(A)** Phenotypes of a 45-day-old transgenic plant expressing 35S-*GhBRC1-A11* (GH_A11G0062) to a mutant *brc1*-2, of a 40-day-old transgenic plant expressing 35S-*GhTCP13* (GH_A05G3929) to a mutant *brc1*-2, and of a 40-day-old transgenic plant expressing 35S-*GhBRC2-A12* (GH_A12G1898) to a mutant *brc1*-2. **(B)** Quantitative analysis of rosette-leaf branches in 45-day-old *brc1*-2 and transgenic plant. **(C)** Number of branches of 10 individual 45-day-old WT, *brc1*-2 plants, and transgenic *Arabidopsis* plants (**p < 0.01). Data represent the mean ± SD from three biological replicates.

*AtBRC1* and *AtBRC2* both arrest axillary bud development and prevent axillary bud outgrowth, but the *brc2* mutant phenotype was weaker than *brc1* (Aguilar-Martínez et al., 2007). *GhBRC1-A11* and *GhBRC2-A12*, the homologues of *AtBRC1* and *AtBRC2*, were selected to rescue the *brc1* mutant phenotype. As expected, *GhBRC1-A11* overexpression in the *brc1* mutant seriously decreased the rosette-leaf branch number compared with the *brc1* mutant: there was an average of eight rosette-leaf branches in the *brc1* mutant, but only about four rosette-leaf branches in the 35S-*GhBRC1-A11*-*brc1*-2 lines. Similarly, *GhBRC2-A12* also restored the *brc1* mutant phenotype, and there were about five rosette-leaf branches in 35S-*GhBRC2-A12*-*brc1*-2 plants (**Figure 5**). Ectopically expressing *GhTCP13* also rescued the *brc1* mutant phenotype (**Figure 5A**). Like *GhBRC1* and *GhBRC2*, *GhTCP13* also belongs to the TCP class II subfamily, shares the conversed TCP domain, and interacts with *GhTIE1* to repress bud activity, supporting the likely essential role of *GhTCP13* in regulating bud activity and shoot branching. The possible explanation was that overexpression of *GhTCP13* in the *brc1*-2 mutant significantly promoted the protein accumulation of *GhTCP13*. *AtTIE1* may mainly interact with *GhTCP13*, weakening the interaction of *AtTIE1* and *AtBRC1* and increasing the activity of *AtBRC1* and expression of *AtHB21*, *AtHB40*, and *AtHB53* to inhibit bud activity. Alternatively, an increased *GhTCP13* transcript level in 35S-*GhTCP13*-*brc1*-2 plants may directly promote *GhHB21* transcription, as shown in **Supplementary Figure 5**; where *GhHB21*pro-LUC and 35S-*GhTCP13* were co-infiltrated, the activity of *GhHB21*pro-LUC was increased.

To study the function of *GhBRC1* in regulating cotton branch number, two copies of the *GhBRC1* gene including GH_A11G0062 and GH_D11G0067 were simultaneously silenced using VIGS. This did not result in differences in the branch number between the silenced *GhBRC1* plants and CLCrV:00 plants (the control line with the empty vector). The possible explanation was that there were four copies of *GhBRC1* (**Supplementary Figure 3**) that showed low similarity excluding the TCP domain with *AtBRC1*. Only two copies including GH_A11G0062 and GH_D11G0067 were silenced in our study. The other two copies of *GhBRC1* (GH_A12G2854 and GH_D12G2879) and the two copies of *GhBRC2* and *GhTCP13* were not simultaneously silenced, which may play a redundant function in regulating branch number.

### *HB21*, *HB40*, and *HB53* Are Negatively Related to Shoot Branch Number in Cotton and *Arabidopsis*

**Figure 6:**
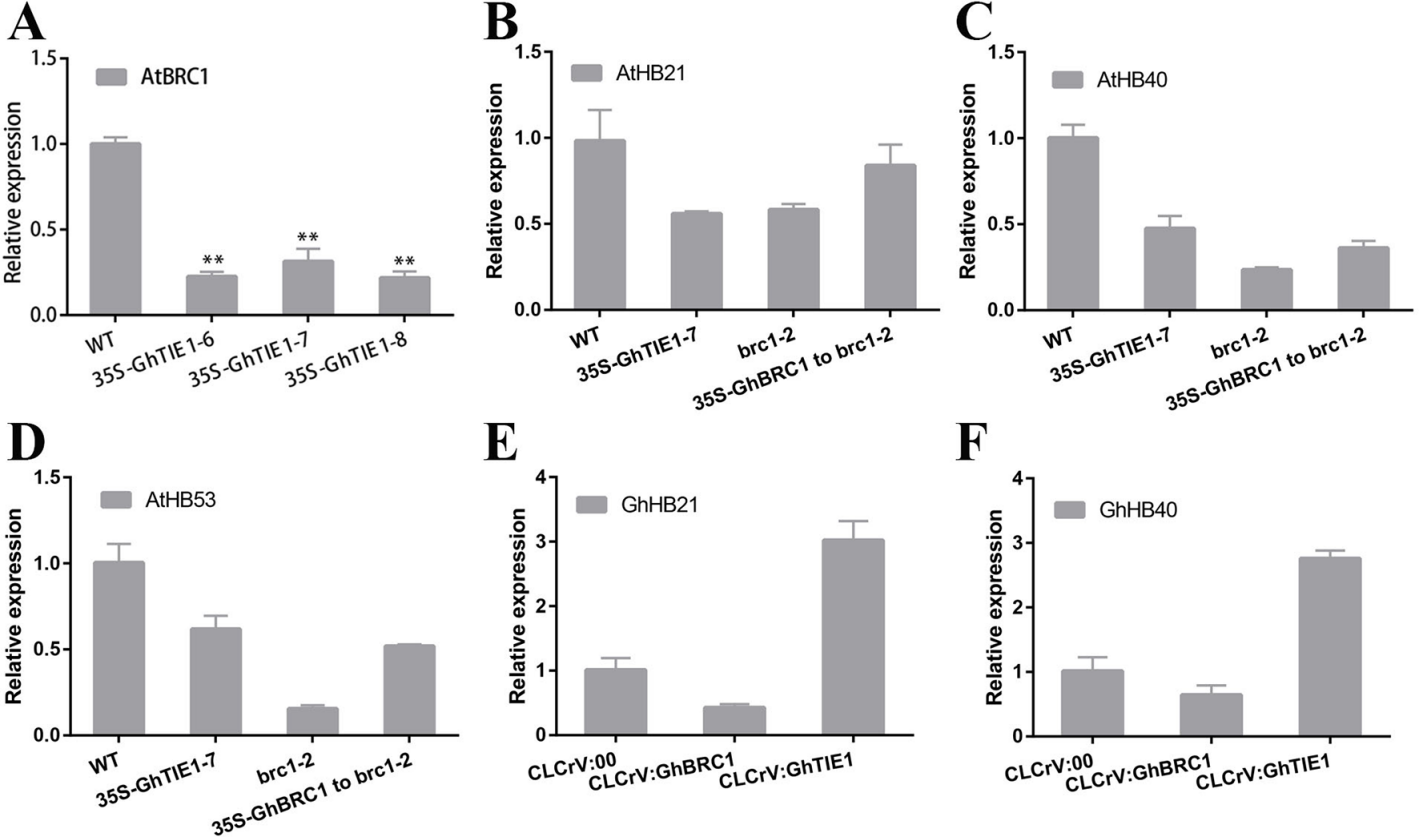
*HB21*, *HB40*, and *HB53* expression levels are inhibited by *TIE1* and *BRC1*. **(A)** *AtBRC1* expression level in wild type (WT) and *GhTIE1*-overexpressing lines. **(B)** *AtHB21* expression levels in WT, *GhTIE1*-overexpressing plant, and *brc1-2* mutant and *GhBRC1* overexpression in *brc1-2* mutant plants. **(C)** *AtHB40* expression levels in WT, *GhTIE1*-overexpressing plant, and *brc1-2* mutant and *GhBRC1* overexpression in *brc1-2* mutant plants. **(D)** *AtHB53* expression levels in WT, *GhTIE1*-overexpressing plant, and *brc1-2* mutant and *GhBRC1-A11* overexpression in *brc1-2* mutant plants. **(E)** *GhHB21* (GH_A02G1904) expression levels in CLCrV:00, CLCrV : GhBRC1, and CLCrV : GhTIE1 plants. **(F)** *GhHB40* (GH_D11G1980) expression levels in CLCrV:00, CLCrV : GhBRC1, and CLCrV : GhTIE1 plants. Each reaction was performed with three biological replicates. Data represent the mean ± SD from three biological replicates.

*HB21*, *HB40*, and *HB53* in *Arabidopsis* are directly regulated by *AtBRC1*, and *AtTIE1* also indirectly repressed the transcription of these genes (Yang et al., 2018). Our hypothesis was that the transcriptional levels of these genes are also closely related to shoot branching number in cotton. Overexpression of *GhTIE1* in *Arabidopsis* significantly increased the shoot branching number, including cauline-leaf branch and rosette-leaf branch, where *AtBRC1*, *AtHB21*, *AtHB40*, and *AtHB53* transcriptions were more seriously inhibited than in WT (**Figures 6A–D**), suggesting that *GhTIE1* may also directly or indirectly regulate *AtBRC1* and three *AtHB* genes at the transcriptional level. *GhBRC1* partially rescued the *brc1*-2 mutant phenotype and the shoot branching number was decreased compared to the *brc1*-2 mutant, but higher than that of the WT. Coinciding with the shoot branching phenotype, *AtHB21*, *AtHB40*, and *AtHB53* expression levels were slightly higher in 35S-*GhBRC1*-*brc1* plants than in the *brc1* mutant (**Figures 6B–D**), showing that the expression of these three genes was negatively related to shoot branch number.

*GhTIE1* was functionally conserved in regulating shoot branching in *Arabidopsis* and cotton. Silencing *GhTIE1* obviously decreased the branch number in cotton. Considering that *HB21*, *HB40*, and *HB53* played important roles in regulating shoot branching, homologous genes in cotton were analyzed. Based on the phylogenetic analysis, only *GhHB21* and *GhHB40* were identified. As expected, *GhHB21* and *GhHB40* were both unregulated in *GhTIE1*-silenced cotton plants, but slightly decreased in *GhBRC1*-silenced lines (**Figures 6E, F**), showing that the expression levels of these *HB* gene were positively regulated by *GhBRC1* but negatively regulated by *GhTIE1*. Additionally, the expression levels of *GhBRC1* and *GhBRC2* were also regulated by *GhTIE1*. *GhBRC1* showed an upregulated expression in *GhTIE1*-silenced plants compared to the control, while *GhBRC2* expression level is similar to the control (**Supplementary Figure 8**), in link with the results of TIE1 function in controlling *BRC1* expression in *Arabidopsis* (Yang et al., 2018).

## Discussion

### *GhTIE1a* and *GhTIE1d* Are Functionally Conserved in Regulating Shoot Branching in Cotton and *Arabidopsis*

Although the plant architecture between *Arabidopsis* and cotton is different, the shoot branch growth was controlled by bud activity in both species. *AtTIE1* positively regulated shoot branching in *Arabidopsis*. Gain-of-function mutant tie1-D and overexpressing *AtTIE1* in *Arabidopsis* produced significantly more branches (Tao et al., 2013). Two homologs of *AtTIE1* in cotton, *GhTIE1a* and *GhTIE1d*, were also essential in regulating bud activity. Overexpression of *GhTIE1a* or *GhTIE1d* in *Arabidopsis* obviously increased the shoot branch number, including rosette-leaf branch and cauline-leaf branch, which presented a similar phenotype to tie1-D. Silencing the conversed sequence between *GhTIE1a* and *GhTIE1d* inhibited the bud activity, and shoot branch number was significantly decreased in CLCrV : *GhTIE1* compared to CLCrV:00 plants, showing that *GhTIE1* is functionally conserved in regulating shoot branching in plants. Silencing *GhTIE1* generated fewer branches than the control. The *VGhTIE1* plants appear to be younger than the wild type. The possible explanation is that there is a balance between vegetative and reproductive growth. Similar results were reported in *Arabidopsis* that disruption of TIE1 causes defects of shoot branching and axillary bud development; that is to say, TIE1-mEAR-VP16 plants produce fewer branches and appear to be younger than the wild type (Yang et al., 2018). Different from *Arabidopsis* architecture, including rosette-leaf branch and cauline-leaf branch, cotton is an infinitely growing crop with a complex branching pattern consisting of fruit branches and nutritional branches, which were developed from the cotton main stem (Chen et al., 2015). Decreased fruit branch number in silenced *GhTIE1* plants partially inhibited the infinite growth, which facilitated the fiber maturation of later cotton bolls and improvement of cotton fiber length uniformity.

### *GhTIE1* as a Transcriptional Repressor Represses GhTCPs Activity *via* Direct Interaction

TCP activity is regulated at the protein level and transcription level and is essential in controlling plant development (Tao et al., 2013). Based on the TCP domain features, TCPs were divided into two classes, class I and class II (three subclass: CIN, CYC, and TB1) (Gaudin et al., 2014; Yin et al., 2018). Yeast two-hybrid screening revealed that *GhTIE1* physically interacted with multiple *GhTCP* proteins, including *GhTCP18* (*GhBRC1*), *GhTCP12* (*GhBRC2*), and *GhTCP13*, and these *GhTCPs* were both grouped into class II. *GhTCP18* and *GhTCP12* were categorized into *TB1* and *CYC*-like TCPs, respectively. *GhTCP13* was categorized into *CIN*-like TCPs (Li et al., 2017). In *Arabidopsis*, *TIE1* not only regulated leaf development *via* suppressing *CIN*-like TCP activity (Tao et al., 2013) but also controlled shoot branching by repressing *BRC1* (*TB1*-like TCP) activity (Yang et al., 2018). Although there was no interaction between *AtTIE1* and *AtBRC2 in vitro*, the *brc2* mutant showed a similar branching phenotype to the *brc1* mutant in *Arabidopsis*, suggesting that *AtBRC2* activity may also be regulated directly or indirectly by *AtTIE1*. Different from *Arabidopsis*, *GhTIE1* and subclass II TCPs (*GhBCR1*, *GhBCR2*, and *GhTCP13*) have strong interactions *in vivo* and *in vitro*, and these TCPs both partially rescued the phenotype of *brc1* mutant in *Arabidopsis*. Previous researches found that CIN-type genes limit cell proliferation at the margins of the developing leaf primordia and regulate leave morphology in *Antirrhinum*, *Arabidopsis*, and tomato (Martín-Trillo and Cubas, 2009; Koyama et al., 2010). Different from the reported function of CIN-type genes, our research found that *GhTCP13* was preferentially expressed in the leaves and axillary buds (**Supplementary Figure 9**), and *GhTCP13* interacted with *GhTIE1* and partially restored the *brc1* mutant phenotype. These results supported that *GhTCP13* may play a key role in regulating leaf and axillary bud development. Considering the reduced lateral branching phenotype caused by overexpression of *GhTCP13* in the *brc1* mutant, a possible explanation is that ectopically expressed *GhTCP13* derived by the 35S promoter was ubiquitously expressed in the whole plant, which may cause the decreased branch number in the *brc1* mutant, suggesting that *GhTCP13* may partially function redundantly in regulating branching with *AtBRC1*, not *AtTCP13*. Alternatively, *GhTCP13* may exhibit a dual function in controlling leaf and axillary bud development in cotton and function differently with *AtTCP13*.

These results show that these TCPs may be functionally redundant in controlling cotton branching, and the regulatory network involved in branching mediated by *TIE1* and TCPs varied in different species.

### *GhTIE1* and *GhBRC1* Regulate Shoot Branching Depending on *HB21*, *HB40*, and *HB53* Expression Levels

Bud dormancy genes *HB21*, *HB40*, and *HB53* were directly regulated by *BRC1* and also modulated by *TIE1* in *Arabidopsis* (Yang et al., 2018). These genes increased the ABA level to inhibit bud activity (Yao and Finlayson, 2015). Our results showed that *GhTIE1* overexpression in *Arabidopsis* promoted more branches, along with the downregulated *AtHB21*, *AtHB40*, and *AtHB53* compared to WT. *GhBRC1* overexpression in the *brc1*-2 mutant decreased the shoot branch number, along with the increased *AtHB21*, *AtHB40*, and *AtHB53* expression levels than in the *brc1*-2 mutant, showing that shoot branching controlled by *GhTIE1* and *GhBRC1* in *Arabidopsis* partially depended on the expression levels of *AtHB21*, *AtHB40*, and *AtHB53*. As expected, silenced *GhTIE1* increased *GhHB21* and *GhHB53* expression, leading to a decreased branch number. Notably, *AtBRC1* was downregulated in *GhTIE1*-overexpressed lines in *A. thaliana*, suggesting that *GhTIE1* may also modulate directly or indirectly *AtBRC1* at the transcriptional level. Meanwhile, *AtBRC1* activity may be repressed by *GhTIE1 via* a direct interaction. The transcriptional and post-transcriptional regulation of *AtBRC1* by *GhTIE1* controlled the expressions of *AtHB21*, *AtHB40*, and *AtHB53*. Contrary to *GhTIE1*, silenced *GhBRC1* repressed *GhHB21* and *GhHB40* transcription, but there were no obvious changes in branch number compared to the control. The possible reason was that *GhBRC1*, *GhBRC2*, and *GhTCP13* were functionally redundant in regulating cotton branching, and *GhBRC2* and *GhTCP13* may compensate for the effect mediated by silenced *GhBRC1*. Genetic compensation was mediated by homologous genes in plants and animals. *AtCLV1* paralogs compensate for the loss of *CLV1* through transcriptional upregulation (Nimchuk et al., 2015). *SlCLE9*, the closest paralog of *SlCLV3*, contributes to stem cell homeostasis by compensating for the loss of *SlCLV3* through transcriptional upregulation in tomato (Rodriguez-Leal et al., 2019). These results proved that the *TIE1*-*BRC1*-*HB21*/*HB40*/*HB53* pathway was conserved in modulating bud activity and shoot branch growth in plants. More efforts should be made to reveal the molecular machinery that controls shoot branching *via* modulation of the activity of the *TIE1* and *TCP* proteins, which will help us to further determine plant shoot architecture in response to environmental cues.

## Data Availability Statement

All datasets generated for this study are included in the article/**Supplementary Files**.

## Author Contributions

XG conceived the original screening and research plan. YangD, JZ, PL, and WH performed the experiments. YZ, LL, XW, YanpD, and PW provided technical assistance to YangD and analyzed the data. XG, YangD and MS wrote the manuscript with all the authors.

## Funding

This research was financially supported by the National Science Foundation of China (31621005, 31701476).

## Conflict of Interest

The authors declare that the research was conducted in the absence of any commercial or financial relationships that could be construed as a potential conflict of interest.
