# Supplementary material for: GhTIE1 Regulates Branching Through Modulating the Transcriptional Activity of TCPs in Cotton and Arabidopsis
Source: Front Plant Sci. 2019 Oct 28;10:1348. doi: 10.3389/fpls.2019.01348 (PMC6827420; doi:10.3389/fpls.2019.01348)
Supplement: Supplementary file 1 [file Table_1.doc]

**Supplementary Table 1**. The primers list used in this study.

| Primer name | Primer sequences (5’-3’) |
| --- | --- |
| QBV3-*GhTIE1d*-F | CAAAAAAGCAGGCTCAGGGGATATC ATGGGTAGCA GCTATTTTGG |
| QBV3-*GhTIE1d*-R | AAAGCTGGGTGCAGGGCGATATC CAATGACAGTCTGCTCCAAATC |
| QBV3-*GhTIE1a*-F | CAAAAAAGCAGGCTCAGGGGATATC ATGGGTAGCG GCTATTTTGG |
| QBV3-*GhTIE1a*-R | AAAGCTGGGTGCAGGGCGATATC CAATGACAGTCTGAGCTCCAAATC |
| 35S-*GhTIE1d*-F | AGAACACGGGGGACTCTAGAATGGGTAGCAGCTATTTTGG |
| 35S-*GhTIE1d*-R | TAGTCAGGCGCGCCGGTACCCAATGACAGTCTGAGCTCCA |
| 35S-*GhTIE1a*-F | AGAACACGGGGGACTCTAGA ATGGGTAGCG GCTATTTTGG |
| 35S-*GhTIE1a*-R | TAGTCAGGCGCGCCGGTACC CAATGACAGTCTGAGCTCCAAATC |
| 35S-*GhBRC1-D11*-F | AGAACACGGGGGACTCTAGA ATGTTTCCTT CAAACAGCAA TGG |
| 35S-*GhBRC1-D11*-R | TAGTCAGGCGCGCCGGTACC CCAAAGGACTGAGCCTGTAAGC |
| 35S-*GhBRC2-D12*-F | AGAACACGGGGGACTCTAGA ATGTTTCCTT CCAGCAACAG TTAC |
| 35S-*GhBRC2-D12*-R | TAGTCAGGCGCGCCGGTACC TAAGTAAAAATGGTGGAATTTGC |
| 35S-*GhTCP13*-F | AGAACACGGGGGACTCTAGA ATGATTCCAG GTTCAGAAGA AG |
| 35S-*GhTCP13*-R | TAGTCAGGCGCGCCGGTACC CTTAGAAGGAAACTCATCTTC |
| V*GhTIE1*-F | CAAAATGGCATGCCTGCAGACTAGTCGTCATCAACTACCTCAGC |
| V*GhTIE1*-R | GAATTCACTAGACCTAGGGGCGCGCCGACAGTCTGAGCTCCAAATC |
| V*GhBRC1*-F | CAAAATGGCATGCCTGCAGACTAGTC GAGAGAAAGAGCAAAGGCAAG |
| V*GhBRC1*-R | GAATTCACTAGACCTAGGGGCGCGCC CCAAAGGACTGAGCCTGTAAGC |
| BD-*GhTIE1*-F | CATATGGCCATGGAGGCC GAATTC ATGGGTAGCA GCTATTTTGG |
| BD-*GhTIE1*-R | TGCGGCCGCTGCAG GTCGAC CAATGACAGTCTGCTCCAAATC |
| AD-*GhBRC1-A11*-F | GCCATGGAGGCCAGT GAATTC ATGTTTCCTT CAAACAGCAA TGG |
| AD-*GhBRC1-A11*-R | TGCAGCTCGAGCTCGAT GGATCCC CCAAAGGACTGAGCCTGTAAGC |
| AD-*GhBRC1-D11*-F | GCCATGGAGGCCAGT GAATTCATGTTTCCTTCAAACAGCAATG |
| AD-*GhBRC1-D11*-R | TGCAGCTCGAGCTCGAT GGATCCCCCAAAGGATTGAGCCTGTAAGC |
| AD-*GhBRC1-A12*-F | GCCATGGAGGCCAGT GAATTCATGTATCCTT CAAACAGCAA TGGC |
| AD-*GhBRC1-A12*-R | TGCAGCTCGAGCTCGAT GGATCCC CTAAATTTTATTGTAGCC |
| AD-*GhBRC1-D12*-F | GCCATGGAGGCCAGT GAATTCATGTATCCTTCAAACAGCAATGG |
| AD-*GhBRC1-D12*-R | TGCAGCTCGAGCTCGAT GGATCCCTTTGTTATAATTAGGTTTGGTTC |
| AD-*GhBRC2-A12*-F | GCCATGGAGGCCAGT GAATTCATGTTTCCTT CCAGCAACAG TTAC |
| AD-*GhBRC2-A12*-R | TGCAGCTCGAGCTCGAT GGATCCCTAAGTAAAAATGGTGGAATTTGC |
| AD-*GhBRC2-D12*-F | GCCATGGAGGCCAGT GAATTCATGTTTCCTTCCAGCAACAGTTAC |
| AD-*GhBRC2-D12*-R | TGCAGCTCGAGCTCGAT GGATCCCTAAGTAAAAATGGTGGAATTTAC |
| AD-*GhTCP13*-F | GCCATGGAGGCCAGT GAATTC ATGATTCCAG GTTCAGAAGA AG |
| AD-*Gh*TCP13-R | TGCAGCTCGAGCTCGATGGATCCC CTTAGAAGGAAACTCATCTTC |
| P106-*GhTIE1*-F | CATCGAGGACGCCGGC GGATCC ATGGGTAGCA GCTATTTTGG |
| P106-*GhTIE1*-R | GAAAGCTCTGCAGGTCGACTCTAGA CAATGACAGTCTGCTCCAAATC |
| P104-*GhBRC1-A11*-F | CAATTACAGGTACCCGG GGATCCATGTTTCCTT CAAACAGCAA TGG |
| P104-*GhBRC1-A11*-R | CTGCCACCGCCGTCGACTCTAGA CCAAAGGACTGAGCCTGTAAGC |
| P104-*GhBRC1-D11*-F | CAATTACAGGTACCCGG GGATCCATGTTTCCTTCAAACAGCAATG |
| P104-*GhBRC1-D11*-R | CTGCCACCGCCGTCGACTCTAGA CCAAAGGATTGAGCCTGTAAGC |
| P104-*GhBRC1-A12*-F | CAATTACAGGTACCCGG GGATCCATGTATCCTT CAAACAGCAA TGGC |
| P104-*GhBRC1-A12*-R | CTGCCACCGCCGTCGACTCTAGA CTAAATTTTATTGTAGCC |
| P104-*GhBRC1-D12*-F | CAATTACAGGTACCCGG GGATCCATGTATCCTTCAAACAGCAATGG |
| P104-*GhBRC1-D12*-R | CTGCCACCGCCGTCGACTCTAGA TTTGTTATAATTAGGTTTGGTTC |
| P104-*GhBRC2-A12*-F | CAATTACAGGTACCCGG GGATCCATGTTTCCTT CCAGCAACAG TTAC |
| P104-*GhBRC2-A12*-R | CTGCCACCGCCGTCGACTCTAGA TAAGTAAAAATGGTGGAATTTGC |
| P104-*GhBRC2-D12*-F | CAATTACAGGTACCCGG GGATCC ATGTTTCCTTCCAGCAACAGTTAC |
| P104-*GhBRC2-D12*-R | CTGCCACCGCCGTCGACTCTAGA TAAGTAAAAATGGTGGAATTTAC |
| P104-*GhTCP13*-F | CAATTACAGGTACCCGG GGATCC ATGATTCCAG GTTCAGAAGA AG |
| P104-*GhTCP13*-R | CTGCCACCGCCGTCGACTCTAGA CTTAGAAGGAAACTCATCTTC |
| pro-*GhHB21*-F | TACAAAAAAGCAGGCTCCGCGGCCGC GTTTACATATGGCATGTAC |
| pro-*GhHB21*-R | GTACAAGAAAGCTGGGTCGGCGCGCC GCTAAGAGAAGTGGATATG |
| q*GhTIE1*-F | GCGTCATCACCTTCCTCATCGT |
| q*GhTIE1*-R | TCGGGGTTCCAACTTGTTGTCG |
| q*GhBRC1*-F | GAACCGTGGAATGGCTACTCGT |
| q*GhBRC1*-R | GACATCCGAGACCGAAGTGGTG |
| q*GhHB21*-F | GCTCGAGGAAGAGTATAGC |
| q*GhHB21*-R | CAGCCTCTCGAAGTTGGTCC |
| q*GhHB40*-F | ATGCCGACTTGAATCCGAGGTG |
| q*GhHB40*-R | CTCGTTCCGTCAGTTGCTGGAT |
| *GhHistone3*F | TCAAGACTGACTGATTTGCGTTT |
| *GhHistone3*R | GCGCAAAGGGTTGGTGTCTTC |
| *Actin*F | AGAAACCCTCGTAGATTGGCAC |
| *Actin*R | ACTCTCCCGCTATGTATGTCGC |
| q*AtHB21*-F | AACCGCTGGATCCAACTCAG |
| q*AtHB21*-R | TTGACGAGGGTCAAGCCCTA |
| q*AtHB40*-F | AGATGGAGGCAACGGTTTGT |
| q*AtHB40*-R | TCCAACCAACTCTTTCCGCA |
| q*AtHB53*-F | GTACCCCTACACGACCCAAC |
| q*AtHB53*-R | CTCCTTCCTCCCTGACTCCA |
| q*AtBRC1*-F | GATTTGACAAAGCCAGCAAAAC |
| q*AtBRC1*-R | TCTTGTTTCGGTCGTGTTAGTA |
| qGhBRC1-A11-F | TCATTTCCCTTCTTCTCCG |
| qGhBRC1-A11-R | CATCAAAGCGTTGTTGTTGTT |
| qGhBRC1-D11-F | GAACCGTGGAATGGCTACTCGT |
| qGhBRC1-D11-R | CACCACTTCGGTCTCGGATGTC |
| qGhBRC1-A12-F | AGGGAAAAGGCAAGAGCAAGGG |
| qGhBRC1-A12-R | TTGTTGTGGTGGTGGTGTTGGA |
| qGhBRC1-D12-F | AGGGAAAAGGCAAGAGCAAGGG |
| qGhBRC1-D12-R | TTGTTGTGGTGGTGCTGTTGGA |
| qGhBRC2-A12-F | GGCATCACAAGATCGTCAGCCA |
| qGhBRC2-A12-R | TGCTCTTGCTCATTAGGGTCCG |
| qGhBRC2-D12-F | ATACTGCGGACTCGGACTCGAT |
| qGhBRC2-D12-R | TGACGATCTTGTGATGCCCAGG |

**Supplementary Table 3**

Statistics of the number of branches in twenty different CLCrV:00 and GhTIE1-silenced cotton plants.

|  | **The number of cotton lateral branches** | | | | | | | | | | | | | | | | | | | |
| --- | --- | --- | --- | --- | --- | --- | --- | --- | --- | --- | --- | --- | --- | --- | --- | --- | --- | --- | --- | --- |
| **ClCrV:00** | **15** | **11** | **10** | **10** | **13** | **13** | **13** | **12** | **12** | **14** | **13** | **16** | **13** | **14** | **14** | **14** | **13** | **11** | **14** | **11** |
| **ClCrV:GhTIE1** | **9** | **10** | **11** | **8** | **10** | **7** | **8** | **9** | **9** | **9** | **6** | **9** | **9** | **6** | **7** | **8** | **7** | **10** | **7** | **8** |


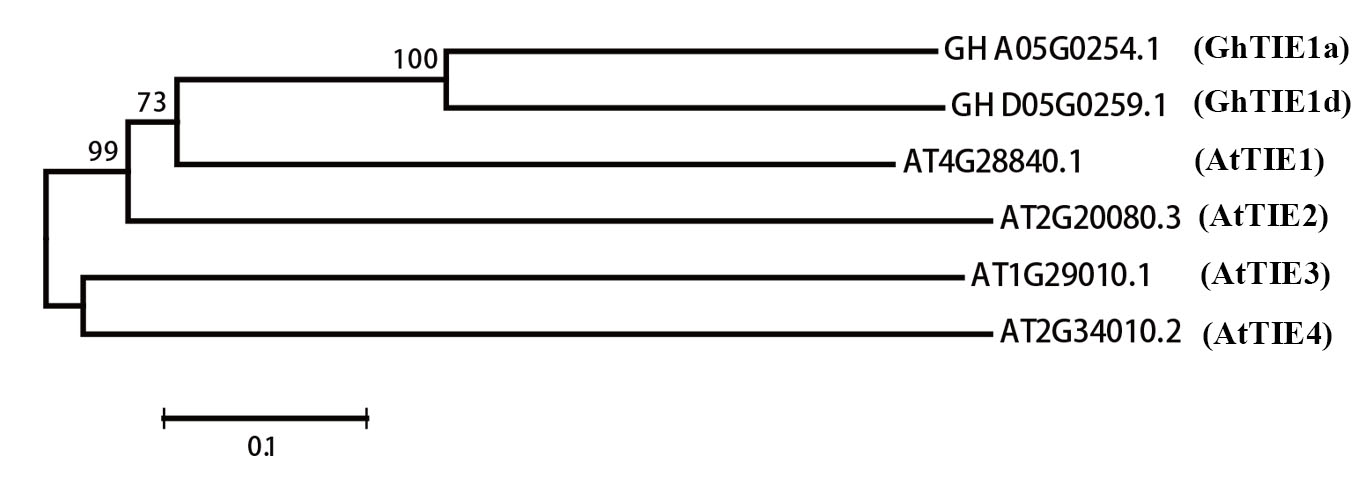


**Supplementary** **Figure 1**

Phylogenetic analysis of GhTIE1 with AtTIE1, AtTIE2, AtTIE3, and AtTIE4.


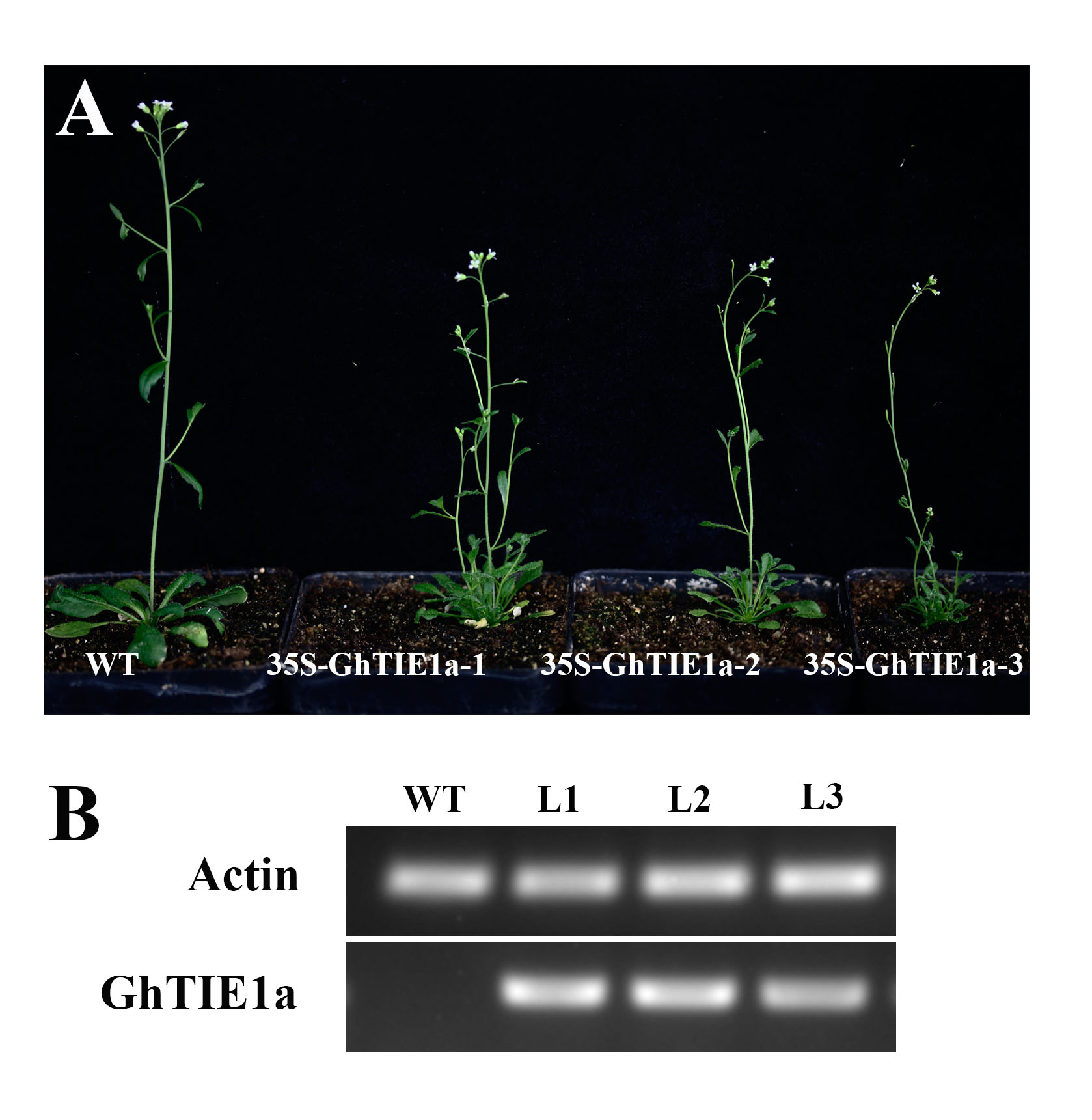


**Supplementary** **Figure** **2**

(**A**) Branching phenotypes of 35-day-old WT plants and three overexpressed 35S-*GhTIE1a*(GH_A05G0254) lines, overexpressing *GhTIE1a* in *Arabidopsis* produce more branches than wild-type plant.(**B**) *GhTIE1a* was not expressed in WT but was highly expressed in three overexpressed lines based on semi-quantitative PCR


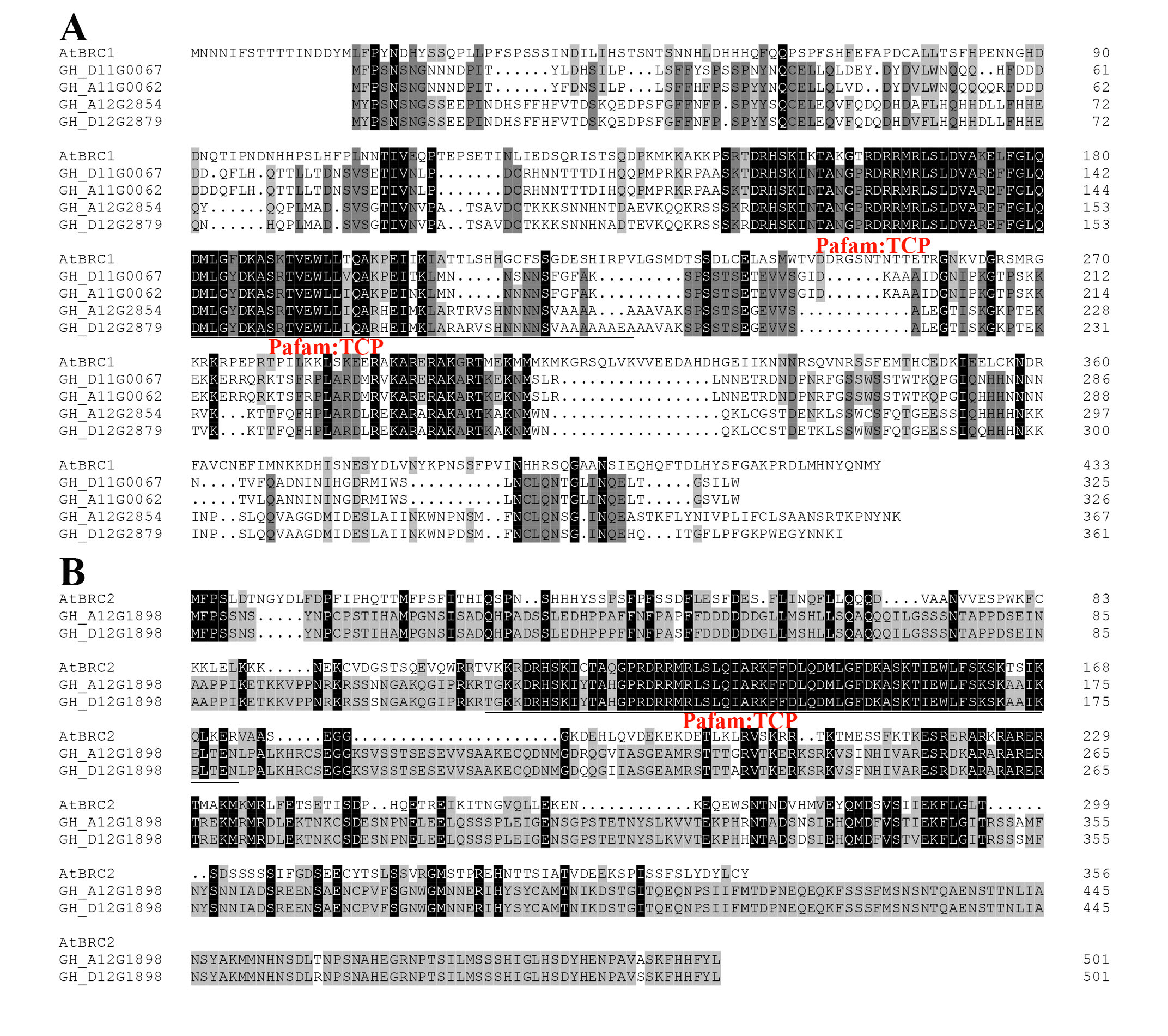


**Supplementary** **Figure** **3**

(**A**) Sequence alignment and domain analysis of *GhBRC1*(GH_A11G0062, GH_D11G0067, GH_D12G2879, GH_A12G2854) in *Arabidopsis* and upland cotton. (**B**) Sequence alignment and domain analysis of *GhBRC2* (GH_A12G1898, GH_D12G1898) in *Arabidopsis* and upland cotton.


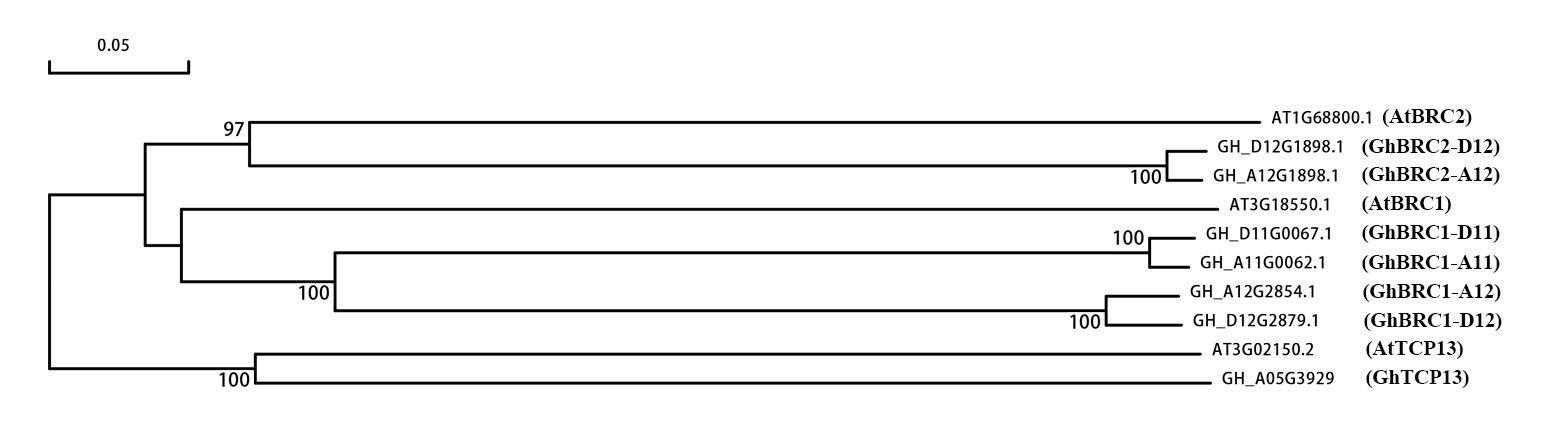


**Supplementary** **Figure 4**

Phylogenetic analysis of GhBRC1, GhBRC2 and GhTCP13 with AtBRC1, AtBRC2, and AtTCP13.


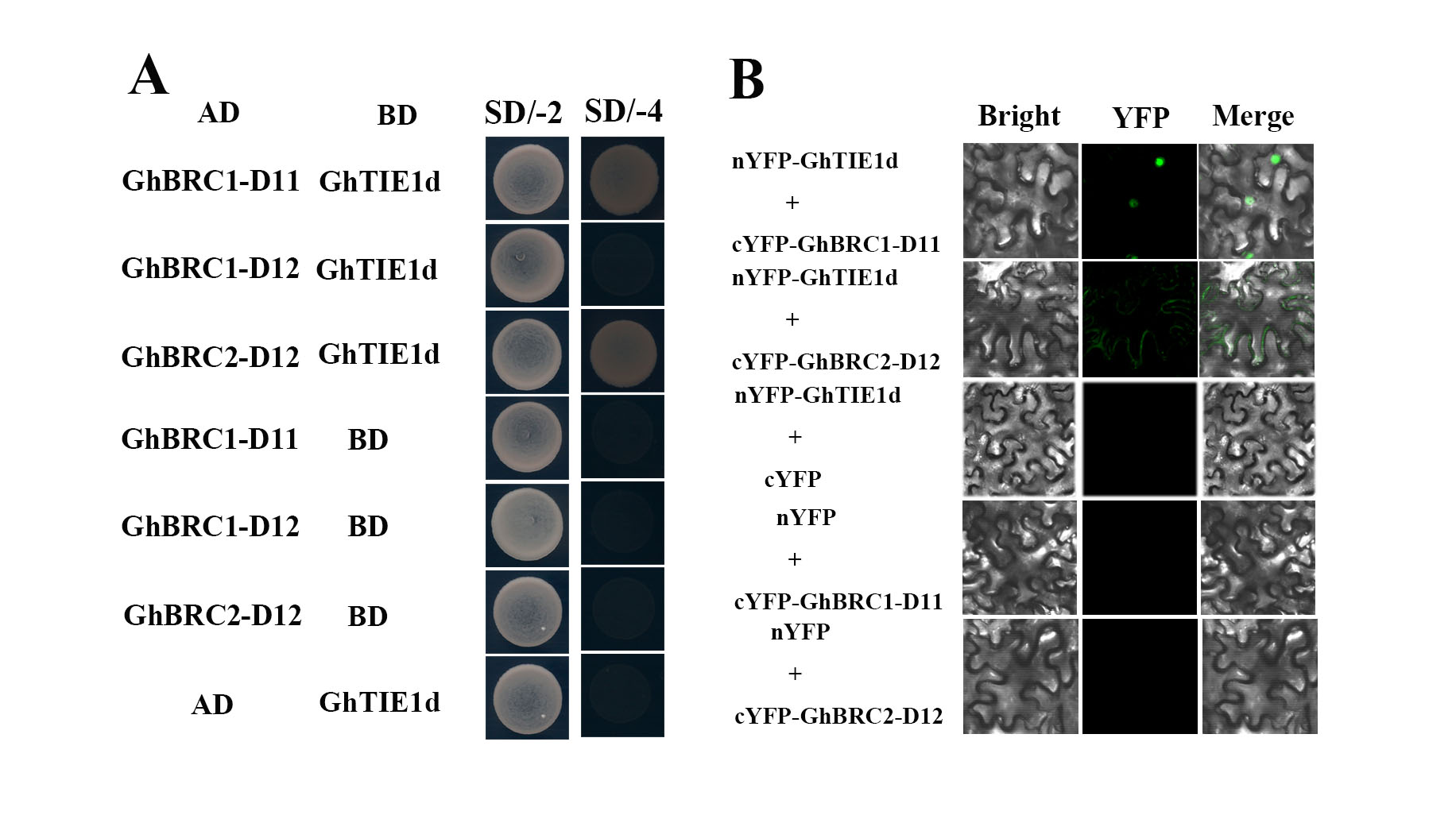


**Supplementary** **Figure 5**

(**A**) Yeast-two-hybrid assays of *GhTIE1* (GH_D05G0259) with *GhBRC1-D11* (GH_D11G0067), *GhBRC1-D12* (GH_D12G2898), *GhBRC2-D12* (GH_D12G1898). (**B**) BiFC assay proved the interaction of *GhTIE1* with *GhBRC1-D11* and *GhBRC2-D12*.


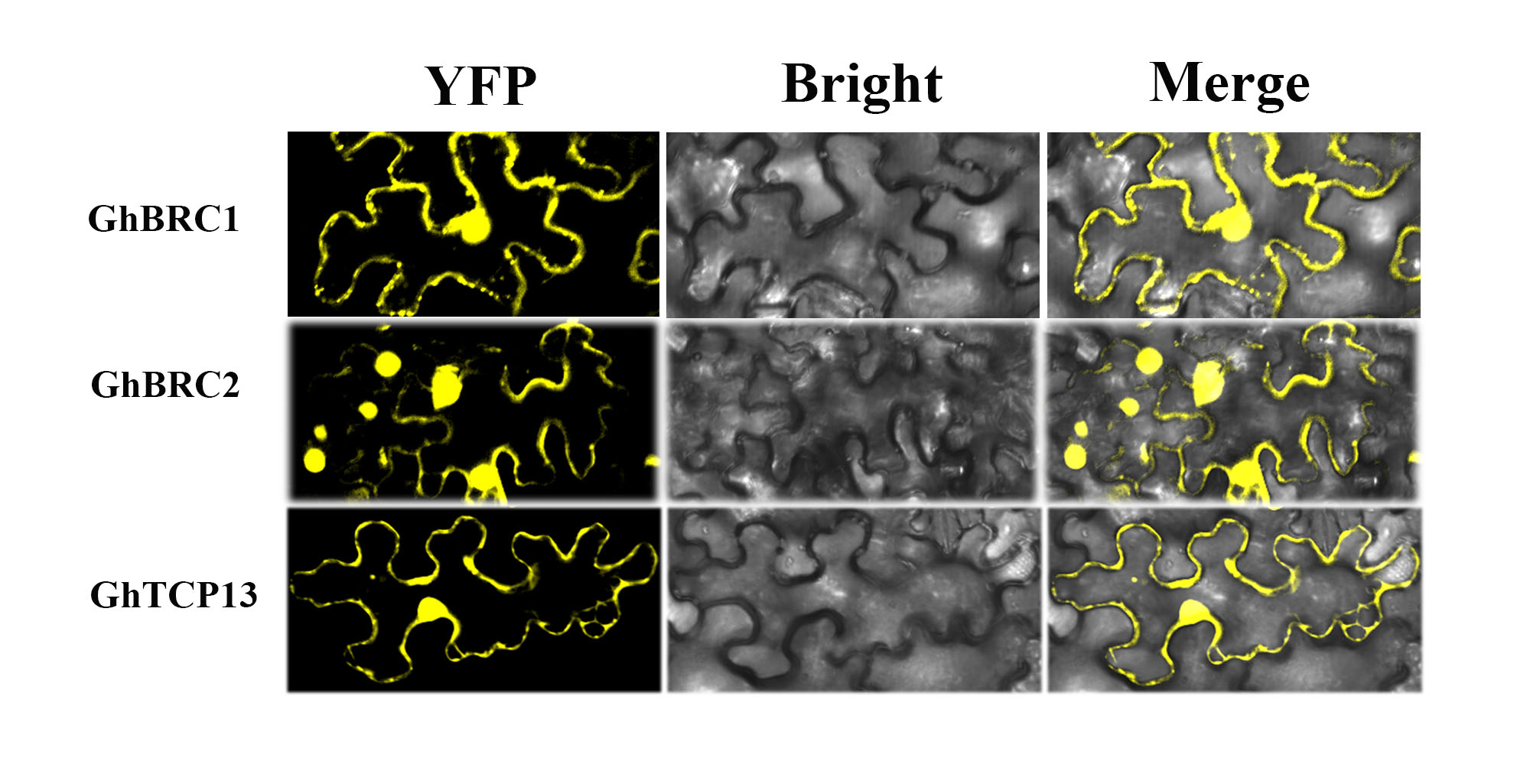


**Supplementary** **Figure 6**

*GhBRC1* (GH_D11G0067), *GhBRC2* (GH_A12G1898) and *GhTCP13* (GH_A05G3929)are localized in the nucleus and cell membrane.


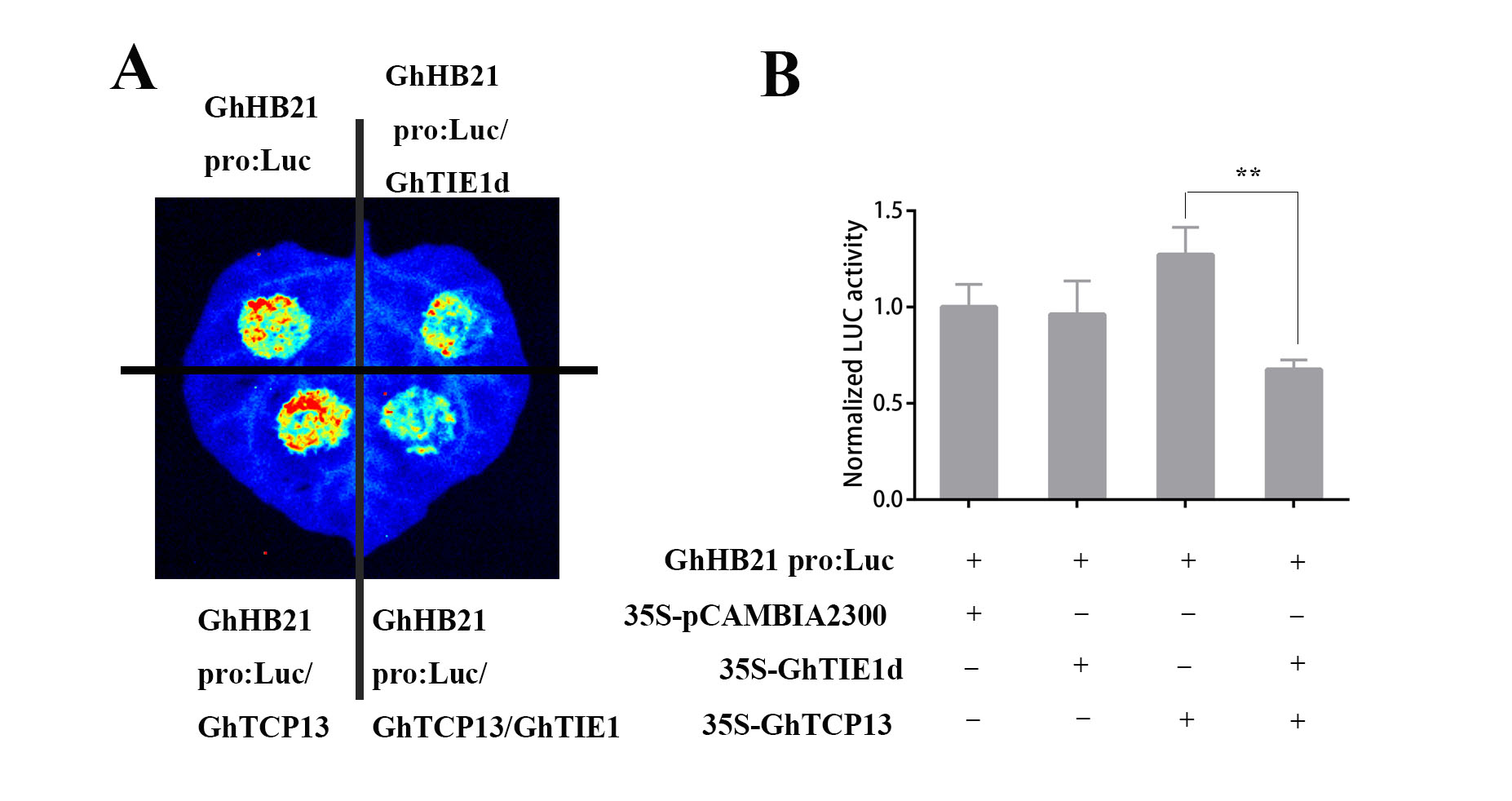


**Supplementary** **Figure 7**

(**A**) The promoter of the *GhHB21* gene fused to LUC was used as a reporter for the transactivation assay, *GhTIE1* inhibited *GhHB21* expression via interaction with *GhTCP13*. (**B**) LUC activity was decreased when *GhTIE1*, *GhTCP13*, and *GhHB21* pro:Luc were co-infiltrated into *N. benthamiana* leaves.


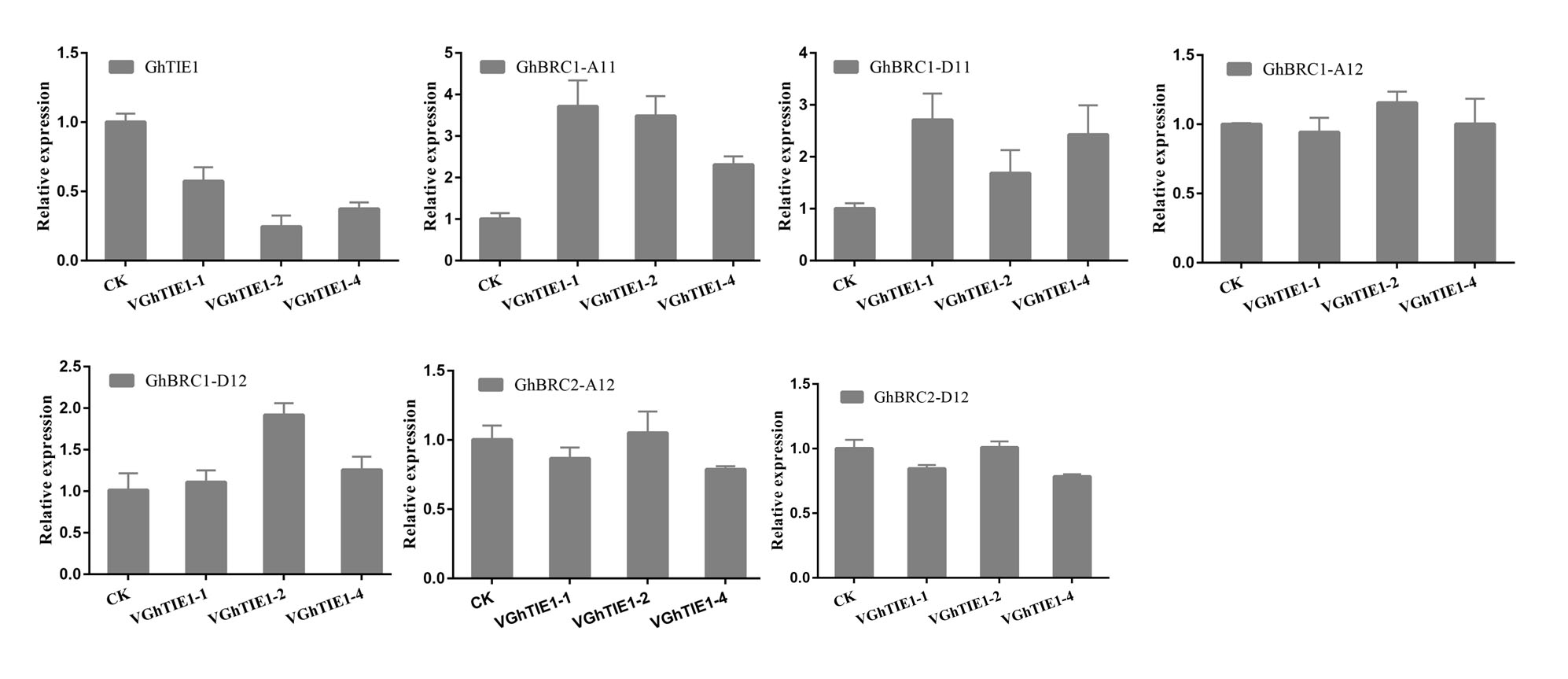


**Supplementary** **Figure 8 The expression level of GhBRC1 and GhBRC2 genes in CLCrV:GhTIE1 line.**

Expression levels of four copies of GhBRC1 were evaluated in CLCrV:GhTIE1 line, while expression level of two copies of GhBRC2 were slightly downregulated.


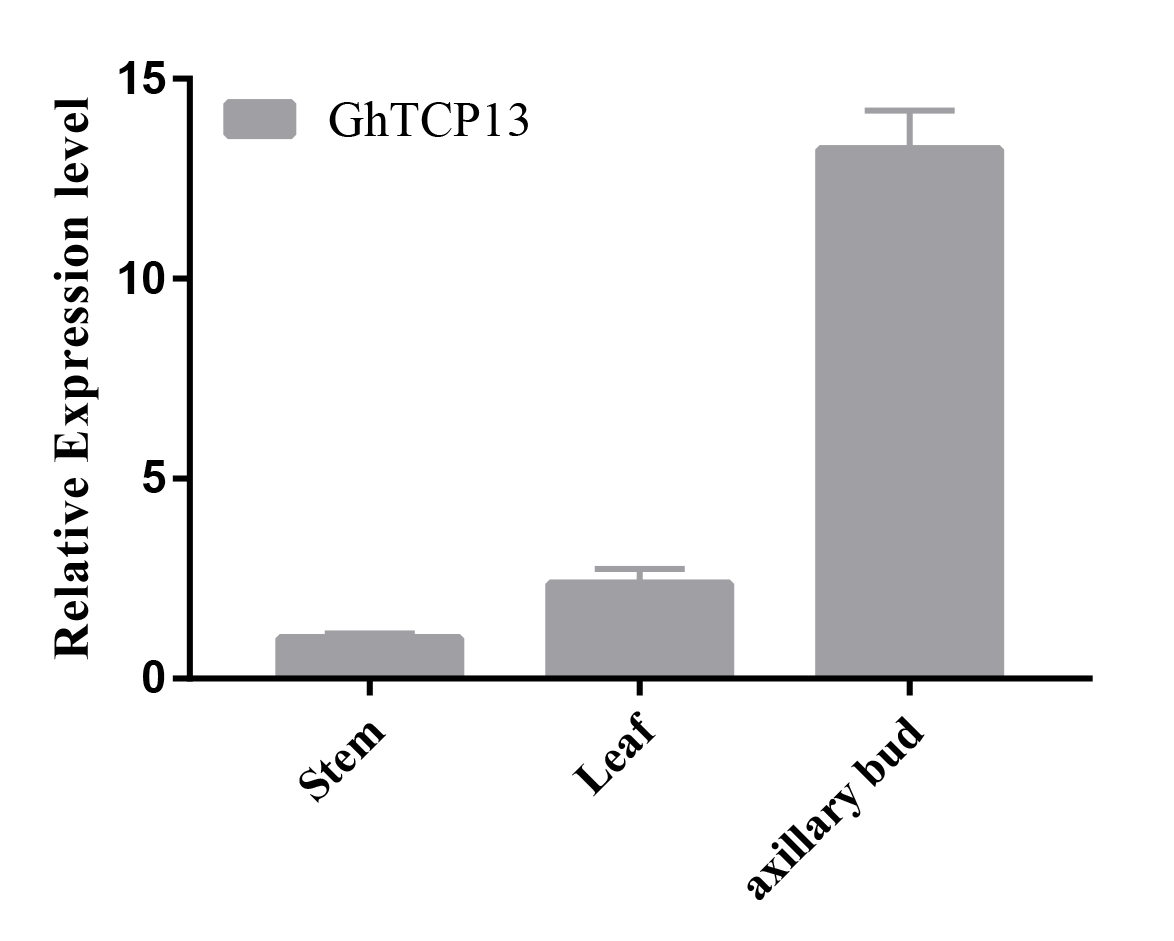


**Supplementary** **Figure 9** Analysis of GhTCP13 expression level in stem, leaf, and axillary bud.
